# Dithiolate auxiliary ligands as electronic modulators in ruthenium-based sensitizers: a DFT and TD-DFT study

**DOI:** 10.1039/d6ra01663d

**Published:** 2026-07-02

**Authors:** Zohreh Abdollahi, Sepideh Samiee, Zabiollah Mahdavifar

**Affiliations:** a Department of Chemistry, Faculty of Science, Shahid Chamran University of Ahvaz Ahvaz Iran z_mahdavifar@scu.ac.ir +98-611-3331042

## Abstract

Rational ligand engineering provides an efficient route for tailoring the electronic structure and photoinduced properties of ruthenium-based sensitizers. In this study, density functional theory (DFT) and time-dependent DFT (TD-DFT) calculations are employed to systematically investigate a series of Ru(ii) dyes with the general formula [Ru(dcbpy)(NCS)_2_(L)], where L represents different dithiolate auxiliary ligands (dcdmp, dmit, pdt, and tdt). The impact of ligand electronic characteristics on frontier molecular orbitals, excited-state composition, optical absorption, and interfacial charge-transfer behavior is comprehensively analyzed. The results show that dithiolate ligands induce significant stabilization of the HOMO and narrowing of the HOMO–LUMO gap compared to the reference dye, thereby enhancing low-energy metal-to-ligand and ligand-to-ligand charge-transfer contributions. Among the investigated complexes, Ru-dcdmp exhibits the smallest energy gap (1.83 eV in the gas phase) and the most favorable energetic alignment for spontaneous electron injection into the TiO_2_ conduction band. TD-DFT simulations reveal broadened and intensified absorption in the visible and near-infrared regions, with solvent-induced enhancement of oscillator strengths in acetonitrile. Explicit adsorption modeling on a (TiO_2_)_8_ cluster confirms strong dye–TiO_2_ interactions, efficient electronic coupling, and effective charge delocalization from the Ru center toward the TiO_2_ surface upon excitation. Most complexes exhibit a bidentate adsorption mode, whereas the Ru-dmit complex adopts a more asymmetric interfacial binding geometry. Overall, this work establishes clear structure–property relationships and highlights dithiolate ligands, particularly dcdmp, as effective electronic modulators for designing high-performance ruthenium sensitizers for dye-sensitized solar cell applications.

## Abstract 2

Rational ligand engineering provides an efficient route for tailoring the electronic structure and photoinduced properties of ruthenium-based sensitizers.

## Graphical abstract

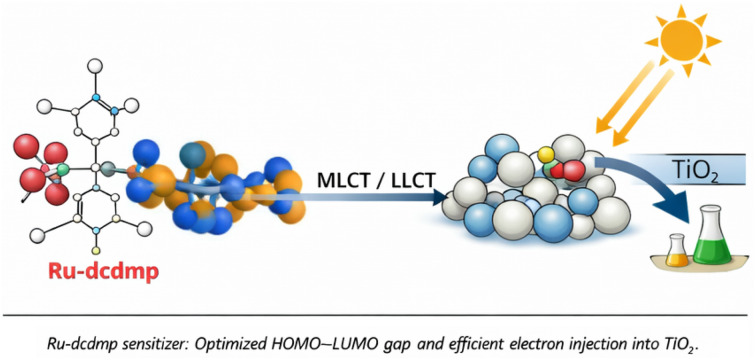

## 1. Introduction

Over the past two decades, dye-sensitized solar cells (DSSCs) have emerged as a versatile platform for investigating photoinduced charge-transfer processes at organic–inorganic interfaces, while simultaneously offering a cost-effective alternative to conventional solid-state photovoltaic technologies. Owing to their relatively low fabrication cost, mechanical flexibility, and tunable optoelectronic properties, DSSCs have attracted sustained attention as representative third-generation solar energy conversion devices.^1–6^ A seminal breakthrough in this field was achieved in 1991 by O'Regan and Grätzel, who demonstrated an efficient DSSC based on nanocrystalline TiO_2_ films sensitized by a molecular dye, thereby establishing the fundamental operational principles of this class of devices.^7^ In a typical DSSC architecture, a molecular sensitizer is anchored onto the surface of a wide-band gap semiconductor through specific functional groups. Upon photoexcitation, the dye undergoes a transition to an excited electronic state, followed by ultrafast electron injection into the conduction band of the semiconductor. The oxidized dye is subsequently regenerated by a redox mediator, most commonly the iodide/triiodide (I^−^/I_3_^−^) couple, thus completing the photocatalytic cycle.^8–11^ From a molecular design perspective, the efficiency of this process critically depends on several interconnected factors: (i) strong and broad absorption across the visible and near-infrared regions of the solar spectrum, (ii) favorable energetic alignment between the excited-state dye levels and the semiconductor conduction band, (iii) efficient dye regeneration kinetics governed by the redox potential of the electrolyte, and (iv) long-term chemical and photochemical stability under operating conditions.^9,11–13^

Among the wide range of sensitizers explored to date, Ru(ii) polypyridyl complexes remain among the most extensively studied and successful systems, owing to their intense metal-to-ligand charge-transfer (MLCT) transitions, tunable redox properties, and exceptional photostability.^14–17^ Despite achieving power conversion efficiencies exceeding 11% in optimized configurations, conventional ruthenium-based dyes often suffer from limited absorption in the long-wavelength region and incomplete utilization of the solar spectrum.^7,18–24^ Consequently, rational ligand engineering has emerged as a key strategy for extending light absorption, modulating frontier orbital energies, and enhancing charge-transfer characteristics.^20–22^ Previous DFT and TD-DFT studies have demonstrated that substituent and acceptor engineering in Ru(ii) polypyridyl sensitizers can significantly modulate frontier orbital distribution, charge-transfer characteristics, and electron-injection energetics, thereby providing an effective strategy for ligand-directed optimization of DSSC performance.^25,26^ Furthermore, previous theoretical and spectroscopic investigations have shown that the low-energy absorption bands in Ru-based chromophores predominantly originate from MLCT excitations with relatively low to moderate oscillator strengths due to the intrinsic nature of the involved electronic transitions.^27,28^ In addition, the excited-state behavior and photophysical properties of polypyridyl Ru complexes are strongly influenced by charge-transfer relaxation pathways and spin–orbit coupling effects, which play an important role in intersystem crossing processes and excited-state dynamics.^29^^.^ These findings highlight the importance of understanding structure–property relationships for the rational design and optimization of Ru-based sensitizers with improved optical and electronic characteristics.

In this context, the incorporation of auxiliary ligands with strong electron-donating or π-conjugated character has proven particularly effective in tailoring the electronic structure of ruthenium sensitizers. Dithiolate ligands, in particular, have attracted growing interest due to their pronounced electron-donating ability and capacity to induce low-energy MLCT and ligand-to-ligand charge-transfer (LLCT) transitions.^23,24,30,31^ By modifying the electronic density around the metal center and altering orbital hybridization, dithiolate ligands offer a powerful means of narrowing the HOMO–LUMO gap and shifting absorption features toward longer wavelengths, thereby improving light-harvesting capability.

**Fig. 1:**
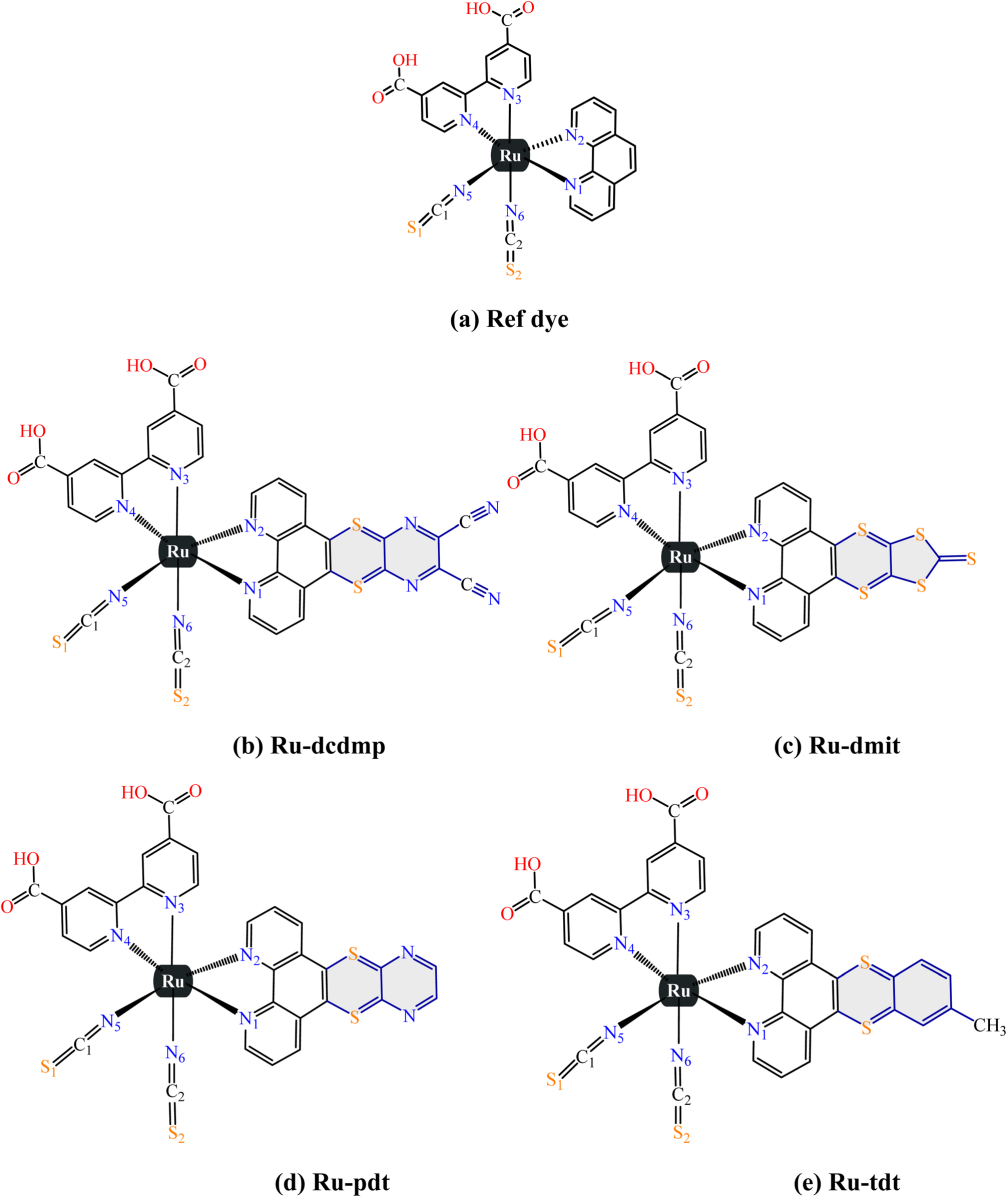
Structures of Ru dyes with different auxiliary ligands.

Motivated by these considerations, the present work focuses on a systematic theoretical investigation of ruthenium sensitizers incorporating dithiolate auxiliary ligands coordinated to polypyridyl frameworks (Fig. 1). Four representative dithiolate ligands, 5,6-dicyano-2,3-dithiolate pyrazine (dcdmp), 1,3-dithiol-2-thione-4,5-dithiolate (dmit), pyrazine-2,3-dithiolate (pdt), and 3,4-toluenedithiolate (tdt), are examined in combination with 4,4′-dicarboxy-2,2′-bipyridine (dcbpy) as the anchoring ligand.^32^ The carboxylate groups of dcbpy ensure robust binding to the TiO_2_ surface, while two thiocyanate (–NCS) ligands coordinated to the Ru center further contribute to fine-tuning the photophysical and electrochemical properties of the complexes. Using density functional theory (DFT) and time-dependent DFT (TD-DFT) calculations, this study aims to elucidate how the electronic characteristics of dithiolate auxiliary ligands influence the frontier orbital distribution, excitation energies, and charge-transfer thermodynamics of the sensitizers, both in the isolated state and upon adsorption onto TiO_2_. By establishing clear structure–property relationships, this work provides molecular-level insight into ligand-driven modulation of ruthenium-based sensitizers, offering theoretical guidance for the rational design of advanced photoactive complexes.

## 2. Computational details

All quantum chemical calculations were performed using the Gaussian 16 program package.^33^ Density functional theory (DFT) calculations was employed to investigate the structural, electronic, and optical properties of ruthenium-based sensitizers incorporating dithiolate auxiliary ligands and carboxylate anchoring groups. Full geometry optimizations were carried out for both isolated dyes and dye@(TiO_2_)_8_ complexes using the hybrid B3LYP exchange–correlation functional,^34,35^ incorporating Grimme's empirical dispersion correction (D3) to properly account for long-range dispersion interactions.^36^ The 6-31G(d,p) basis set was adopted for all non-metal atoms, while the LANL2DZ effective core potential (ECP)^37^ and corresponding basis set were applied to Ru and Ti atoms. This combination of functional and basis sets has been extensively validated in previous studies, including our earlier work, and has proven to provide a reliable description of the geometric and electronic properties of ruthenium dyes and their complexes with TiO_2_.^38–42^

To assess the reliability of the selected theoretical approach, additional benchmark calculations were performed on the well-known N3 dye, for which reliable experimental data are available. Geometry optimizations were carried out using several exchange–correlation functionals, including B3LYP, CAM-B3LYP, WB97XD, PBEPBE, and M06-2X, in ethanol solution (see Tables S1 and S2 in the SI). The results indicate that B3LYP provides the closest agreement with experimental data, particularly for the HOMO–LUMO gap (1.76 eV *vs.* 1.68 eV experimentally) and the Ru–ligand coordination bond lengths. In contrast, the alternative functionals either significantly overestimate the excitation energies or lead to less satisfactory structural parameters. Based on this comparative analysis, we conclude that B3LYP offers the most balanced and reliable description of both the geometric and electronic properties of Ru(ii) polypyridyl systems within the scope of the present work. All optimizations were performed without imposing symmetry constraints. Harmonic vibrational frequency calculations were subsequently conducted at the same level of theory to ensure that the optimized geometries correspond to true local minima on the potential energy surface, as confirmed by the absence of imaginary frequencies. Solvent effects were incorporated using the polarizable continuum model (PCM) within the self-consistent reaction field (SCRF) framework, with acetonitrile employed as the dielectric medium to mimic experimental conditions.^43,44^ To further investigate the electronic properties of the complexes, natural bond orbital (NBO) analysis^45^ was performed at the B3LYP/6-31+G(d,p):LANL2DZ level of theory. These calculations were conducted in both the gas phase and acetonitrile solvent to determine the NBO-derived atomic charges.

Time-dependent DFT (TD-DFT) calculations were performed to characterize the excited-state properties and to simulate the UV-visible absorption spectra of the sensitizers. These calculations were carried out at the same theoretical level as the ground-state optimizations to maintain methodological consistency. For the dye@(TiO_2_)_8_ systems, the dyes were anchored to the TiO_2_ nanocluster through their carboxylate groups, and full geometry optimizations, followed by frequency analyses, were performed prior to evaluating their electronic structure and photoinduced charge-transfer characteristics. The electronic and optical properties of both free and adsorbed dyes were systematically analyzed to elucidate the influence of dithiolate auxiliary ligands and interfacial binding on charge-transfer behavior. Molecular structures, frontier molecular orbitals, and electron density distributions were visualized using GaussView, while simulated absorption spectra were processed with GaussSum. Additional wavefunction-based analyses, including polarization-related properties, were carried out using the Multiwfn program.^46^

### 2.1 Theoretical framework for electronic structure and charge-transfer descriptors

The electronic properties of the investigated systems were analyzed in terms of the energies of the frontier molecular orbitals, namely the highest occupied molecular orbital (HOMO) and the lowest unoccupied molecular orbital (LUMO), as well as the corresponding HOMO–LUMO energy gap (*E*_HLG_). In addition, global reactivity descriptors derived from conceptual density functional theory—including chemical hardness (*η*), softness (*S*), chemical potential (*µ*), and electrophilicity index (*ω*) were evaluated using eqn (1)–(5):

1 *E*_HLG_ = *E*_LUMO_ − *E*_HOMO_

2 *η* = (*E*_LUMO_ − *E*_HOMO_)/2

3 *S* = 1/2*η*

4 *µ* = (*E*_LUMO_ + *E*_HOMO_)/2

5 *ω* = *µ*^2^/2*η*

here, *E*_HOMO_and *E*_LUMO_ denote the energies of the highest occupied and lowest unoccupied molecular orbitals, respectively. These descriptors provide quantitative insight into the electronic stability, charge-accepting capability, and overall reactivity of the dyes and have been widely employed to rationalize structure–property relationships in photoactive systems.^47–52^ To further elucidate charge distribution and donor–acceptor interactions, NBO analysis was performed for both isolated dyes and dye–semiconductor complexes, enabling a detailed examination of intramolecular charge transfer and interfacial electronic coupling.^53^

The light-harvesting efficiency (LHE), which reflects the ability of a sensitizer to absorb incident photons, was estimated from the oscillator strength (*f*) associated with the dominant electronic transition using eqn (6):

6 LHE = 1 − 10^−*f*^

where *f* corresponds to the oscillator strength at a given excitation wavelength.^47^ To characterize photoinduced charge-transfer processes at the dye–semiconductor interface, key energetic parameters were evaluated, including the free energy of electron injection (Δ*G*_inj_), the open-circuit voltage (*V*_oc_), the recombination driving force (Δ*G*_rec_), and the free energy change associated with dye regeneration (Δ*G*_reg_). These quantities were calculated using eqn (7–11):7
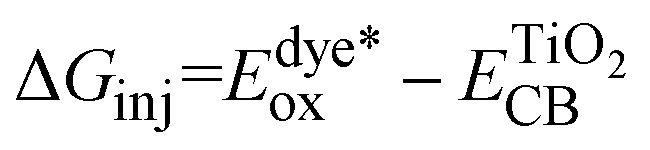

8 *V*_oc_ = *E*_LUMO_ − *E*^TiO^_CB_^_2_^

9
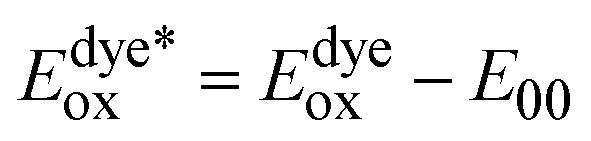

10 Δ*G*_rec_ = *E*^dye^_ox_ − *E*^TiO^_CB_^_2_^

11 Δ*G*_reg_ = *E*^dye^_ox_ − *E*^electrolyte^_redox_

where 
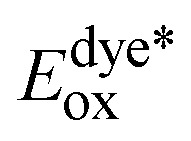
 and *E*^dye^_ox_ represent the excited- and ground-state oxidation potentials of the dye, respectively, *E*_00_ denotes the vertical excitation energy corresponding to the maximum absorption wavelength (*λ*_max_), and *E*^TiO^_CB_^_2_^ is the conduction band edge of TiO_2_.^9,11,54–56^ These energetic descriptors provide a thermodynamic framework for assessing the feasibility of electron injection, recombination, and dye regeneration processes.

Nonlinear optical (NLO) properties play a crucial role in enhancing intramolecular charge transfer (ICT) characteristics and electron delocalization, which leads to improved electron injection efficiency and photocurrent response in DSSCs. The isotropic polarizability (〈*α〉*) and the total first hyperpolarizability (*β*_tot_) were calculated using eqn (12) and (13),^57^ respectively:12
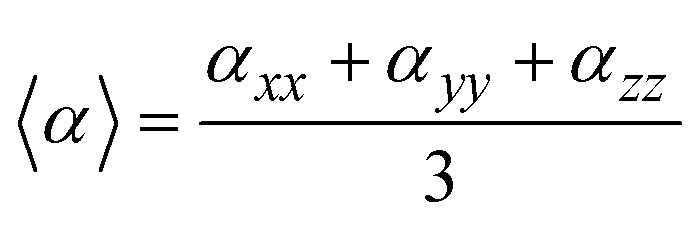
13
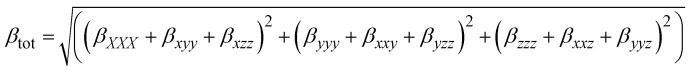


These properties were computed at the B3LYP/6-31G(d,p)/LANL2DZ level in both the gas phase and acetonitrile solvent and serve as sensitive indicators of electronic polarization and nonlinear response in molecular systems. Finally, the adsorption energy (*E*_ads_) of the dyes on the (TiO_2_)_8_ nanocluster was calculated using eqn (14):14
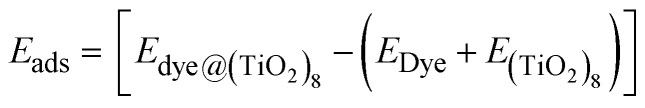
where *E*_dye@(TiO_2_)_8__, *E*_dye_, and *E*_(TiO_2_)_8__denote the total energies of the dye–TiO_2_ complex, the isolated dye, and the bare TiO_2_ cluster, respectively.^53–55^ This parameter provides a quantitative measure of interfacial stability and electronic coupling strength at the dye–semiconductor interface.

## 3 Results and discussion

### 3.1 Geometry optimization and frontier molecular orbitals (FMOs) analysis

The optimized geometrical parameters of the investigated ruthenium complexes were systematically analyzed and compared with the reference dye and available experimental/theoretical data, as summarized in Table 1. To assess the reliability of our computational approach, the optimized structures were compared with literature data for the well known Ru(ii) dye cis di(thiocyanato) bis(2,2′ bipyridyl 4,4′ dicarboxylate)ruthenium(ii) (N3).^58^ This complex has been experimentally synthesized and extensively characterized, serving as a benchmark sensitizer in dye-sensitized systems. Incorporation of dithiolate auxiliary ligands (dcdmp, dmit, pdt, and tdt) induces only minor structural variations around the Ru(ii) coordination center. In particular, the Ru–N bond lengths remain within a narrow range of 2.06–2.14 Å in the gas phase, consistent with experimental values reported for similar Ru(ii) polypyridyl complexes. This agreement is supported by experimental data from a combined experimental-theoretical study of Ru(ii)-polypyridyl sensitizers^59^ For instance, the Ru–N_1_ bond lengths are found to be approximately 2.13–2.14 Å, while Ru–N_3_ distances fall in the range of 2.06–2.07 Å. The calculated bond angles around the ruthenium center show slight deviations from experimental measurements. The N_1_–Ru–N_2_ angle is predicted to lie between 77.4° and 77.8°, marginally lower than the experimentally reported values exceeding 78°.^58,59^ Such deviations can be attributed to the combined steric and electronic effects introduced by the auxiliary dithiolate ligands, as well as the inherent tendency of the B3LYP functional to slightly overestimate bond lengths and compress coordination angles. Furthermore, a comparative analysis of the optimized geometrical parameters in the gas phase and acetonitrile revealed only minor variations in bond lengths and coordination angles, indicating the high structural stability of the Ru-based dyes across different media; the detailed comparison is provided in Table S1 of the SI.

**Table 1:** Calculated structural parameters of the optimized Ru dyes, including selected bond lengths (Å) and bond angles (°), obtained at the DFT/B3LYP/6-31G(d)/LANL2DZ level in the gas phase, together with comparison to available literature data^a^; atom numbering corresponds to Fig. 1

	Ref dye	Ru-dcdmp	Ru-dmit	Ru-pdt	Ru-tdt	Exp^56^	Exp^53^
Ru–N1	2.14	2.13	2.13	2.13	2.13	2.06	2.05
Ru–N2	2.08	2.09	2.09	2.09	2.09	2.03	2.04
Ru–N3	2.06	2.07	2.06	2.06	2.06	2.06	2.05
Ru–N4	2.14	2.14	2.14	2.14	2.14	2.03	—
Ru–N5	2.09	2.09	2.09	2.09	2.09	2.05	—
Ru–N6	2.07	2.07	2.07	2.07	2.07	2.05	—
N5=C1	1.18	1.18	1.18	1.18	1.18	—	1.12
C1=S1	1.64	1.64	1.64	1.64	1.64	—	1.65
N1–Ru–N2	77.87	77.42	77.45	77.43	77.47	79.80	78.70
N1–Ru–N5	94.70	94.17	94.15	94.05	94.09	97.80	97.20
N2–Ru–N5	93.26	94.24	94.09	94.04	93.82	95.90	96.40
N2–Ru–N4	173.41	174.88	174.64	174.53	174.23	174.50	173.00
N3–Ru–N6	90.66	91.34	91.28	91.22	91.11	90.60	90.70

a The last two columns report reference data taken from ref. 56 (combined experimental–theoretical study of Ru(ii)-polypyridyl sensitizers, including N3) and ref. 53 (purely theoretical study), respectively.

Nevertheless, the overall pseudo-octahedral geometry of the Ru(ii) coordination sphere is well preserved across all complexes. This structural stability suggests that the substitution of auxiliary ligands does not compromise the integrity of the coordination environment, which is a key factor for robust anchoring to the semiconductor surface and facilitating efficient charge-transfer processes in DSSC applications.^60^ The balanced influence of the heteroaromatic dithiolate ligands on the molecular geometry highlights their suitability as auxiliary ligands, enabling fine structural tuning without introducing significant distortions. This level of agreement between computed and experimental geometrical parameters supports the reliability of the computational protocol and provides a solid foundation for interpreting the electronic and photophysical properties discussed below.

**Fig. 2:**
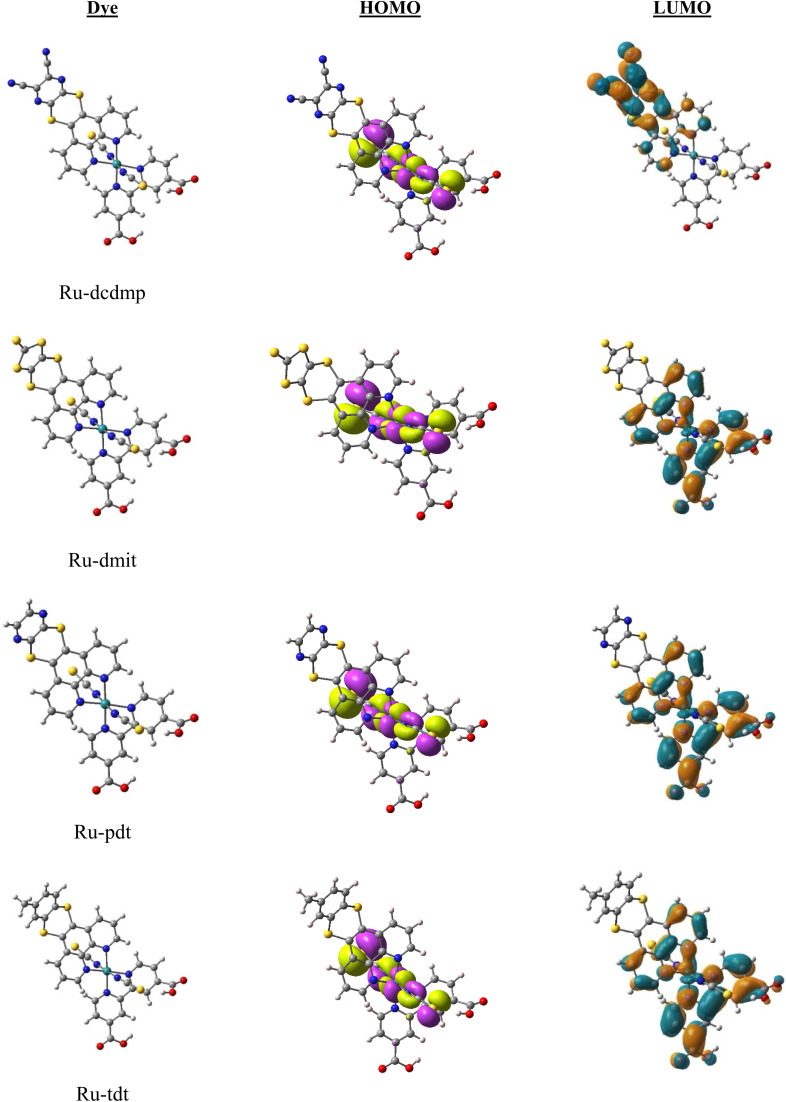
Optimized structures and frontier molecular orbitals (FMOs) of the Ru dyes calculated at the DFT/B3LYP/6-31G(d), LANL2DZ level in the gas phase.

The electronic structures of the dyes were further analyzed through frontier molecular orbital (FMO) calculations, which are particularly relevant for understanding charge-transfer processes in DSSCs. In metal-centered sensitizers, the HOMO and LUMO can be viewed as the primary donor and acceptor states, respectively, forming an efficient intramolecular “push–pull” system that promotes electron injection and suppresses charge recombination. As illustrated in Fig. 2, the HOMO of all investigated complexes is mainly localized on the ruthenium center and the thiocyanate (NCS) ligands, reflecting their dominant role as electron-donating units. In contrast, the LUMO distribution is strongly influenced by the nature of the auxiliary dithiolate ligand. For the complex containing the dcdmp ligand, the LUMO is predominantly localized on the auxiliary ligand itself, indicating a pronounced ligand-centered acceptor character. In the remaining complexes, the LUMO is mainly distributed over the bipyridine framework and the carboxylate anchoring groups, which is highly favorable for electron injection into the conduction band of TiO_2_. This spatial separation between the HOMO and LUMO facilitates efficient intramolecular charge transfer, enhances electron–hole separation, and reduces recombination losses, key factors governing the performance of DSSCs. Moreover, FMO calculations performed in the solvent phase reveal no significant qualitative differences compared to the gas-phase results, indicating that the electronic structure and orbital localization patterns are robust with respect to environmental effects. The corresponding solvent-phase orbital plots are presented in Fig. S1. This consistency further confirms the stability of the electronic features and validates the applicability of the computational approach for predicting structure–property relationships in Ru-based DSSC sensitizers.

### 3.2 Electronic structure and reactivity descriptors

**Fig. 3:**
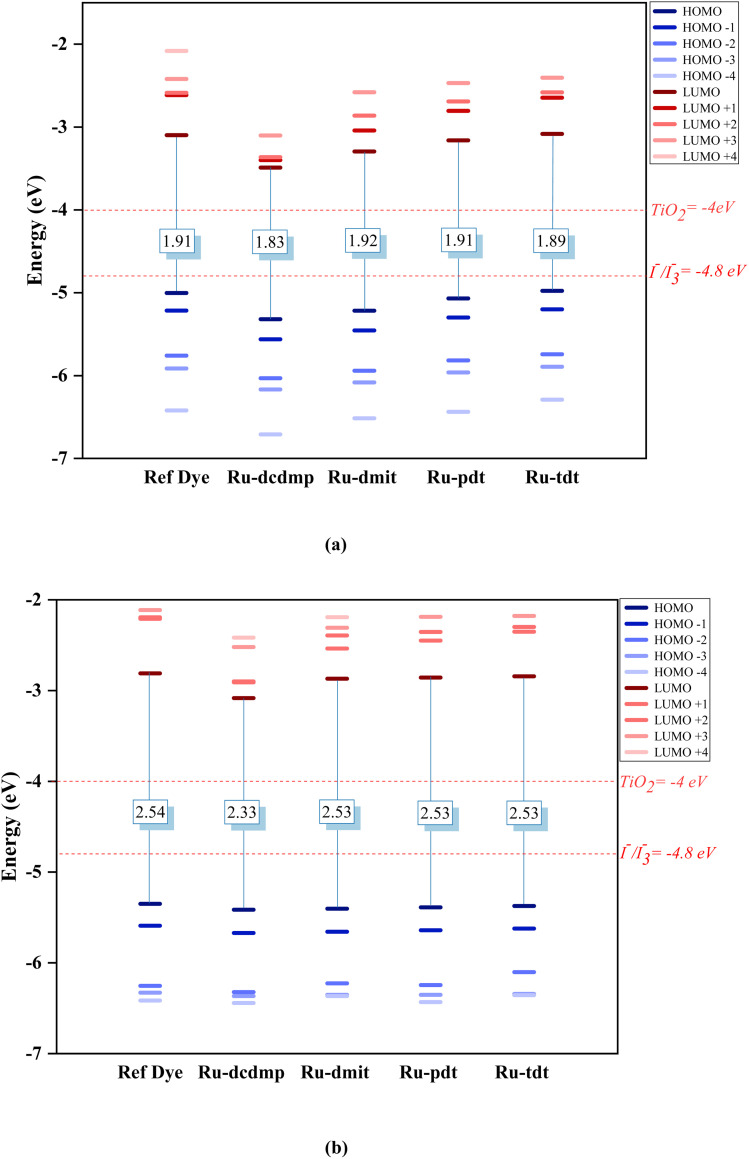
Calculated molecular orbital energy level diagrams of the Ru dyes obtained at the DFT/B3LYP/6-31G(d), LANL2DZ level in (a) the gas phase and (b) acetonitrile solution. The HOMO–LUMO energy gaps are given in eV.

A detailed analysis of the frontier molecular orbital (FMO) energies, including HOMO, LUMO, and the corresponding HOMO–LUMO energy gap (*E*_HLG_), together with key global reactivity descriptors, was carried out for all investigated dithiolate-based dyes in both the gas phase and acetonitrile solvent. The calculated electronic parameters are summarized in Table 2 and graphically illustrated in Fig. 3, providing direct insight into the electronic structure modulation induced by auxiliary dithiolate ligands. As shown in Fig. 3, incorporation of the dcdmp ligand leads to a pronounced stabilization of the HOMO level compared to the reference dye. Specifically, the HOMO energy of Ru–dcdmp decreases from −5.00 eV (reference) to −5.32 eV in the gas phase and from −5.35 eV to −5.41 eV in the solvent phase. This downward shift indicates enhanced stabilization of the oxidized dye state, which is beneficial for suppressing charge recombination and facilitating efficient hole transport during the photoelectrochemical cycle of DSSCs. Regarding the solvation effect, our PCM analysis reveals a differential response between the HOMO and LUMO levels. The HOMO, which is primarily localized on the Ru-d orbitals and the sulfur-containing dithiolate/NCS ligands, exhibits a stronger stabilization in the polar acetonitrile environment due to its higher localized electron density and polar character. In contrast, the LUMO, which is predominantly delocalized over the π* orbitals of the 4,4′-dicarboxy-2,2′-bipyridine (dcbpy) acceptor ligand, exhibits less pronounced stabilization- or even a relative destabilization-compared to the gas phase (*i.e.*, less negative energy values in ACN). This differentiated response to the solvent arises from the distinct orbital compositions: the HOMO, with its higher localized electron density on the Ru-d/dithiolate moiety, interacts more effectively with the polar solvent environment than the diffuse, delocalized π* character of the LUMO. Consequently, the solvation-induced differential shifting of these frontier orbitals leads to an increased HOMO–LUMO gap in the solvent phase, a physical trend that consistently correlates with the calculated electronic excitation energies and the blue-shifted absorption features discussed in Section 3.3. Overall, the incorporation of the dcdmp ligand modulates the frontier orbital energies and maintains a relatively narrow energy gap, which facilitates intramolecular charge transfer (ICT) and promotes efficient photoinduced electron migration from the donor region toward the acceptor anchoring group, thereby supporting effective charge separation in DSSC systems.

**Table 2:** *E*_HOMO_ (*E*_H_), *E*_LUMO_ (*E*_L_), HOMO–LUMO gap energies (*E*_HLG_), chemical potential (*µ*), chemical hardness (*η*), softness (*S*), electrophilicity index (*ω*) isotropic polarizability (*α*) and first hyperpolarizability (*β*) for Ru-dyes (in gas and acetonitrile phases) using the DFT/B3LYP/6-31G(d), LANL2DZ method

	*E*_H_ (eV)	*E*_L_ (eV)	*E*_HLG_ (eV)	*µ* (eV)	*η* (eV)	*S* (eV^−1^)	*ω* (eV)	*α* (a.u.)	*β* (a.u.)
**Gas phase**									
Ref dye	−5.00	−3.09	1.91	−4.05	0.95	0.52	8.61	494.29	9909.41
Ru-dcdmp	−5.32	−3.49	1.83	−4.40	0.91	0.55	10.60	648.64	3664.03
Ru-dmit	−5.22	−3.29	1.92	−4.25	0.96	0.52	9.42	637.63	7159.90
Ru-pdt	−5.07	−3.16	1.91	−4.11	0.95	0.52	8.88	599.74	7579.75
Ru-tdt	−4.98	−3.08	1.89	−4.03	0.95	0.53	8.58	623.27	10 315.43

**Acetonitrile phase**									
Ref dye	−5.35	−2.81	2.54	−4.08	1.27	0.39	6.56	814.04	43 096.31
Ru-dcdmp	−5.41	−3.08	2.33	−4.25	1.17	0.43	7.74	1005.08	16 603.48
Ru-dmit	−5.40	−2.87	2.53	−4.14	1.27	0.39	6.75	1006.09	23 253.51
Ru-pdt	−5.39	−2.86	2.53	−4.12	1.27	0.39	6.71	947.93	25 692.97
Ru-tdt	−5.37	−2.84	2.53	−4.11	1.27	0.39	6.66	980.13	32 339.10

Beyond orbital energies, global chemical reactivity descriptors, including chemical potential (*µ*), chemical hardness (*η*), softness (*S*), and electrophilicity index (*ω*), were evaluated to further elucidate the charge-transfer characteristics of the dyes. The chemical potential of Ru-dcdmp is notably more negative (−4.40 eV) than that of the reference dye (−4.05 eV), reflecting a stronger electron-accepting tendency and enhanced electronic stabilization upon excitation. Meanwhile, the reduced chemical hardness (0.91 eV for Ru-dcdmp *versus* 0.95 eV for the reference) implies increased electronic flexibility and a greater facility for charge redistribution within the molecule. This behavior is closely associated with improved intramolecular charge transport and potentially higher short-circuit current density (*J*_sc_). The electrophilicity index further supports this conclusion, as Ru-dcdmp exhibits a significantly higher *ω* value (10.60 eV) compared to the reference dye (8.61 eV), indicating superior stabilization of the dye upon electron uptake. Collectively, the increased softness and electrophilicity underscore the enhanced ability of Ru-dcdmp to mediate efficient charge separation and transport while minimizing electron–hole recombination losses under illumination. Comparable trends are observed for the Ru-dmit, Ru-pdt, and Ru-tdt dyes, albeit with subtle variations dictated by the electronic nature and conjugation patterns of their respective dithiolate ligands. These ligand-dependent electronic modulations highlight the critical role of auxiliary ligand engineering in fine-tuning the electronic structure and optimizing photoinduced charge dynamics in DSSC sensitizers.^61^

The nonlinear optical (NLO) parameters, including isotropic polarizability (*α*) and first hyperpolarizability (*β*) are well-established descriptors of the molecular charge-transfer capability and intramolecular charge redistribution upon excitation. As shown in Table 2, all dyes exhibit a significant increase in both *α* and *β* in the acetonitrile phase compared to the gas phase. Specifically, *β* values increase by approximately four to five times, reflecting a pronounced solvent-induced enhancement of the intramolecular charge-transfer (ICT) character. While the reference dye displays the highest absolute *β* values, it is observed that the ICT enhancement ratio is comparable across all dyes, including Ru-dcdmp. This suggests that while the reference dye possesses a stronger inherent NLO response, the solvent environment effectively polarizes all molecules, optimizing their electronic redistribution capabilities.

Finally, the energetic alignment of the frontier orbitals with respect to the TiO_2_ conduction band and the electrolyte redox potential was evaluated. The proper alignment of the HOMO and LUMO energy levels relative to the redox potential of the electrolyte and the conduction band edge of TiO_2_ is critical for efficient operation of DSSCs. As shown in Fig. 3, for all investigated dyes, the LUMO energies are sufficiently higher than the TiO_2_ conduction band edge, ensuring spontaneous electron injection from the excited dye. Simultaneously, the HOMO levels lie below the redox potential of the iodide/triiodide electrolyte, enabling efficient dye regeneration after electron transfer. Among the studied systems, Ru-dcdmp exhibits the most favorable energetic alignment combined with the smallest energy gap, reinforcing its superior potential for efficient charge separation, reduced recombination, and enhanced photovoltaic performance.

### 3.3 Optical absorption properties and electronic transitions

The electronic absorption properties of the ruthenium-based dyes incorporating dcdmp, dmit, pdt, and tdt auxiliary ligands were systematically investigated using TD-DFT calculations and compared with the reference dye in both the gas phase and acetonitrile solvent. As summarized in Table S2, the calculated UV-Vis absorption maxima, HOMO–LUMO gaps, oscillator strength (*f*) and light-harvesting efficiency (LHE) obtained using different exchange–correlation functionals were evaluated against the experimental data reported for the N3 dye in ethanol to validate the computational protocol. Although range-separated and meta-hybrid functionals are frequently recommended for charge-transfer excitations, CAM-B3LYP, M06-2X, and ωB97X-D substantially overestimated the excitation energies of the Ru(ii)-based MLCT states for N3, leading to pronounced blue shifts relative to experiment,^62–64^ whereas PBE showed the opposite tendency by underestimating the gap and producing a red shift. In contrast, B3LYP provided the closest agreement with experiment, predicting *λ*_max_ = 393 nm compared with the experimental value of 385 nm (error < 0.1 eV, within the typical accuracy of TD-DFT for transition-metal complexes) together with a band gap of 1.76 eV (experimental: 1.68 eV). Importantly, B3LYP also yields a transition intensity for N3 (*f* = 0.34) comparable to CAM-B3LYP (*f* = 0.31) and ωB97X-D (*f* = 0.41), indicating that it does not systematically underestimate oscillator strengths for the present Ru-based chromophores.

**Fig. 4:**
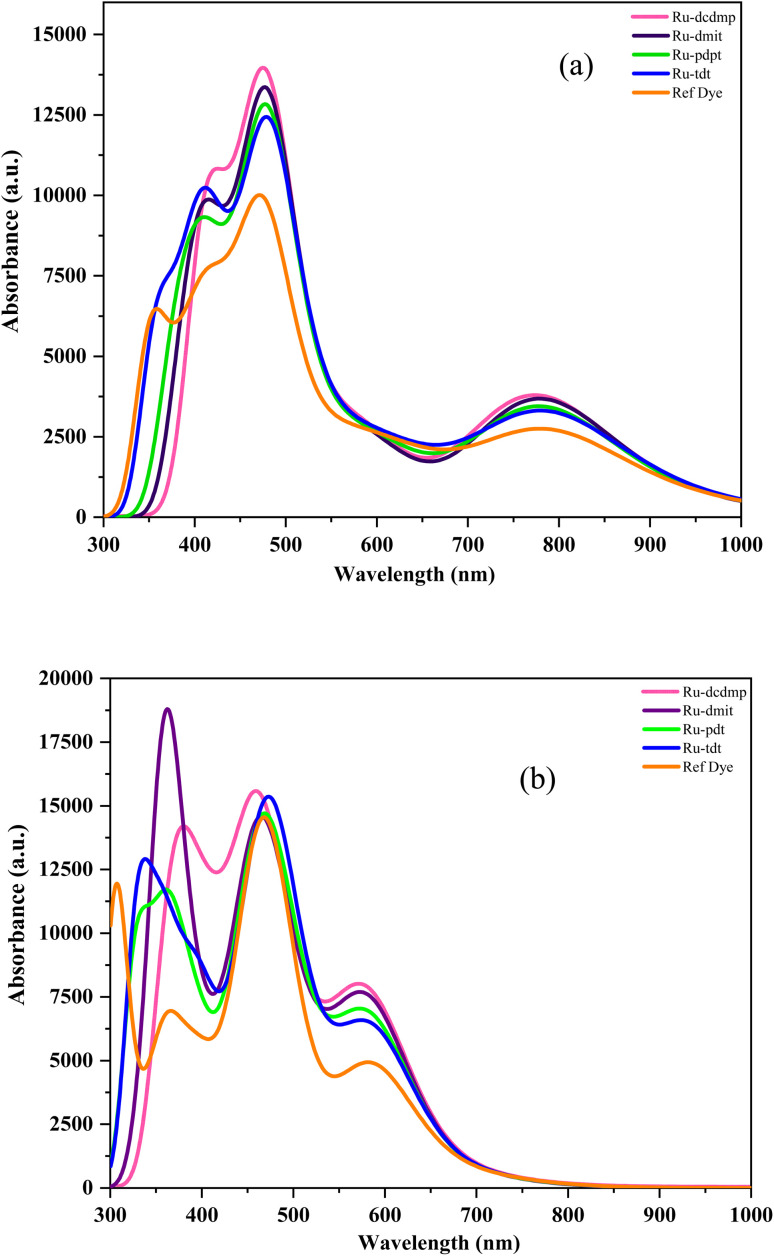
Theoretical UV-visible absorption spectra of the Ru dyes calculated at the DFT/B3LYP/6-31G(d), LANL2DZ level in (a) the gas phase and (b) acetonitrile solution.

The simulated absorption spectra, together with the dominant electronic transitions, oscillator strengths, and orbital contributions, are summarized in Table 3 and depicted in Fig. 4. In the gas phase (Fig. 4a), all investigated dyes exhibit two main absorption regions: a low-energy band extending into the visible to near-infrared (NIR) region (≈780–795 nm) and a higher-energy band located in the visible region between 400 and 500 nm. The long-wavelength absorption feature is assigned to MLCT transitions dominated by excitations from orbitals near the HOMO into the LUMO/LUMO+1 manifold (*e.g.*, H−1 → LUMO or H−1 → LUMO+1), appearing at 795 nm for the reference dye and Ru-tdt, 789 nm for Ru-pdt, and slightly blue-shifted to 781 and 780 nm for Ru-dcdmp and Ru-dmit, respectively. In all cases, the MLCT character is dominant, with donor–acceptor orbital contributions exceeding 88%, confirming efficient charge-transfer pathways from the Ru center/thiocyanate ligands toward the acceptor moieties.

**Table 3:** The absorption energies (*E*_ex_), maximum absorption wavelengths (*λ*_max_), oscillator strength (*f*), major transitions (M.T) of the Ru-dyes (in gas and acetonitrile phases) using the DFT/B3LYP/6-31G(d), LANL2DZ method

Dye	State	*E*_ex_	*λ*_max_	*f*	M.T
**Gas phase**					
Ref dye	2	1.56	795	0.03	H−1 → LUMO (95%)
	14	2.61	475	0.08	H−4 → LUMO (44%), H−1 → L+4 (28%)
Ru-dcdmp	4	1.59	781	0.04	H−1 → L+1 (68%), H−1 → L+2 (15%)
	22	2.57	482	0.12	H−4 → L+1 (56%), H−4 → L+2 (16%), H−1 → L+6 (10%)
	32	2.96	419	0.03	H−6 → L+1 (26%), HOMO → L+10 (30%)
Ru-dmit	3	1.59	780	0.05	H−1 → LUMO (88%)
	16	2.56	484	0.10	H−5 → LUMO (49%), H−4 → LUMO (16%), H−1 → L+5 (18%)
	30	3.01	411	0.04	H−8 → LUMO (34%), HOMO → L+11 (27%)
Ru-pdt	2	1.57	789	0.04	H−1 → LUMO (92%)
	16	2.61	476	0.08	H−4 → LUMO (26%), H−1 → L+5 (56%)
	28	3.03	410	0.02	H−7 → LUMO (17%), HOMO → L+10 (25%)
Ru-tdt	2	1.56	795	0.04	H−1 → LUMO (95%)
	15	2.59	479	0.10	H−4 → LUMO (26%), H−1 → L+4 (47%)
	26	3.04	407	0.02	H−4 → L+1 (47%), H−4 → L+2 (15%)
	35	3.35	370	0.02	H−6 → L+1 (69%), H−6 → L+2 (11%)

**Acetonitrile phase**					
Ref dye	2	2.12	585	0.07	H−1 → LUMO (93%)
	6	2.62	473	0.14	H−2 → LUMO (38%), H−1 → L+1 (41%), HOMO → L+2 (11%)
Ru-dcdmp	3	2.13	582	0.09	H−1 → L+1 (17%), H−1 → L+2 (74%)
	12	2.70	460	0.14	H−2 → L+1 (10%), H−2 → L+2 (46%), HOMO → L+5 (15%)
	29	3.27	379	0.06	H−5 → L+1 (54%), H−2 → L+1 (13%), H−2 → L+5 (11%)
Ru-dmit	2	2.13	582	0.09	H−1 → LUMO (93%)
	8	2.70	459	0.12	H−4 → LUMO (21%), H−3 → LUMO (12%), H−2 → LUMO (23%), H−1 → L+2 (10%), HOMO → L+4 (14%)
	29	3.44	361	0.03	H−4 → L+2 (15%), H−3 → L+2 (10%), H−2 → L+2 (53%)
Ru-pdt	2	2.13	583	0.08	H−1 → LUMO (94%)
	8	2.70	460	0.10	H−2 → LUMO (43%), H−1 → L+2 (15%), HOMO → L+3 (14%)
	26	3.44	361	0.04	H−7 → LUMO (15%), H−4 → L+1 (68%), H−4 → L+2 (11%)
	36	3.77	329	0.09	H−5 → L+4 (28%), H−2 → L+4 (65%)
Ru-tdt	2	2.12	585	0.08	H−1 → LUMO (93%)
	6	2.57	482	0.11	H−4 → LUMO (14%), H−2 → LUMO (13%), H−1 → L+2 (53%)
	30	3.61	343	0.01	H−3 → L+3 (89%)

Despite the favorable positioning of this NIR MLCT band, its oscillator strength (*f*) remains relatively low (*f* ≈ 0.03–0.05), indicating a weak but consistent transition across the dye series. In contrast, significantly stronger absorption features are observed at shorter wavelengths. In particular, pronounced visible bands appear around 480–485 nm, with oscillator strengths reaching 0.12 for Ru-dcdmp (482 nm) and approximately 0.10 for Ru-dmit (484 nm) and Ru-tdt (479 nm). These transitions typically exhibit mixed MLCT/LLCT (ligand-to-ligand charge transfer) character (*e.g.*, H−4 → L+1 or H−5 → LUMO, depending on the auxiliary ligand), reflecting ligand-dependent modulation of the transition dipole moment. Additional weaker absorptions in the near-UV region (≈370–420 nm) are assigned to ligand-centered π → π* transitions. It should be emphasized that the oscillator strengths used for benchmarking in Table S2 correspond to the principal absorption maxima of N3, whereas the smaller oscillator strengths reported in Table 3 are associated with individual low-energy MLCT excitations; for the dominant visible bands in acetonitrile (∼460–480 nm), the computed oscillator strengths (0.10–0.14) are consistent with the broad visible absorption profiles commonly observed for Ru(ii)-polypyridyl sensitizers.^27,28^ Overall, while the position of the lowest-energy MLCT band is only weakly affected by ligand substitution, the auxiliary ligands significantly influence absorption intensity in the visible region, with Ru-dcdmp displaying the highest oscillator strength and consequently the most favorable relative light-harvesting characteristics. Therefore, the computed LHE values are best interpreted as a robust descriptor for comparing relative light-harvesting trends across the investigated dye series rather than as absolute experimental efficiencies. Similar ligand-dependent modulation of MLCT band intensity and spectral position has been widely reported for Ru(ii) polypyridyl sensitizers, confirming that acceptor and substituent engineering can effectively tune the photophysical response while preserving the fundamental MLCT character of the lowest-energy transitions.^65^

The solvent effect plays a crucial role in modulating the optical response of the dyes. Acetonitrile is widely used in dye-sensitized solar cell systems due to its high polarity and aprotic character, which can significantly influence charge-transfer processes. Experimental studies have shown that polar aprotic solvents such as acetonitrile facilitate dye adsorption on TiO_2_ surfaces and promote faster electron injection from the excited dye into the semiconductor, contributing to improved device performance. Moreover, recent investigations have highlighted the key role of MLCT excited states in the photophysical and photochemical behavior of Ru(ii) complexes, including systems containing coordinated acetonitrile, suggesting that the solvent environment can play an important role in modulating MLCT-related processes in Ru-based sensitizers.^66,67^

In acetonitrile (Fig. 4b), the principal MLCT absorption band undergoes a substantial blue shift, relocating from the 780–795 nm region in the gas phase to approximately 582–585 nm. This behavior is consistent with the solvent-induced reorganization of the frontier molecular orbital energies and the corresponding increase in the HOMO–LUMO energy gap in the polar medium, leading to higher transition energies. Importantly, the presence of the solvent significantly enhances the oscillator strength of the MLCT transition, increasing *f* values from ∼0.03–0.05 in the gas phase to ∼0.07–0.09 in acetonitrile. Such enhancement indicates a higher transition probability and more efficient electronic coupling associated with intramolecular charge-transfer processes under realistic DSSC operating conditions. Moreover, the absorption bands in the 460–485 nm region become more intense and better resolved in the solvent environment, with oscillator strengths reaching up to 0.14. These observations suggest that the polar solvent promotes a more favorable electronic distribution and transition dipole moment for charge-transfer excitations while preserving the dominant MLCT character of the excited states. Despite these quantitative changes, the nature of the dominant transitions remains largely unchanged, retaining a strong MLCT character with orbital contributions exceeding 88%. The coexistence of multiple transitions spanning the visible and ultraviolet regions enables broad spectral coverage, which is highly advantageous for maximizing photon harvesting and photocurrent generation in dye-sensitized solar cells.

It should be pointed out that the lowest-energy absorption bands in all complexes arise from MLCT transitions and display relatively small oscillator strengths (*f* = 0.03–0.05 in the gas phase and 0.07–0.09 in acetonitrile), which is consistent with the typical behavior of Ru(ii) polypyridyl sensitizers.^68,69^ These moderate intensities originate from the limited spatial overlap between the Ru-centered dπ donor orbitals and the ligand π* acceptor orbitals, together with the known tendency of B3LYP to underestimate *f* values for d → π* charge-transfer excitations. Although the HOMO–LUMO gaps decrease slightly across the ligand series, this energetic narrowing does not lead to stronger MLCT bands because the oscillator strength is mainly governed by the transition dipole moment rather than by the frontier orbital separation; as the donor–acceptor overlap does not increase in parallel with the reduced gap, the resulting MLCT features remain relatively weak but well defined. Since these MLCT transitions constitute the primary photoactive channels for electron injection in DSSCs, their modest intensities inherently limit the population of excited states capable of transferring electrons to the TiO_2_ conduction band, and therefore are expected to constrain the achievable short-circuit current density (*J*_sc_), despite their favorable energetic alignment.

In summary, the TD-DFT results demonstrate that both auxiliary ligand selection and solvent environment critically govern the optical properties of ruthenium sensitizers. While the long-wavelength MLCT transition is largely conserved across the complexes, the absorption intensity in the visible region is significantly enhanced through appropriate dithiolate ligand engineering, particularly in the case of Ru-dcdmp. The combined effects of solvent-induced orbital reorganization, enhanced oscillator strength, and broad absorption coverage highlight the potential of these dyes for improved light-harvesting efficiency and enhanced photovoltaic performance in DSSC applications.

### 3.4 Photovoltaic parameters and charge-transfer thermodynamics

The key photovoltaic parameters governing the performance of dye-sensitized solar cells, including the open-circuit voltage (*V*_oc_), light-harvesting efficiency (LHE), free energy of electron injection (Δ*G*_inj_), recombination driving force (Δ*G*_rec_), and dye regeneration free energy (Δ*G*_reg_), were systematically evaluated for all investigated ruthenium-based dyes and the reference system. The calculated values in both the gas phase and acetonitrile solvent are summarized in Table 4 and provide valuable insight into the thermodynamic feasibility and efficiency of the individual charge-transfer processes. In the gas phase, all dyes exhibit negative Δ*G*_inj_ values, confirming that electron injection from the photoexcited dye into the TiO_2_ conduction band is thermodynamically spontaneous for all systems. Among the investigated complexes, Ru-dcdmp displays the highest light-harvesting efficiency (LHE = 0.24), indicating its superior capacity to absorb incident photons and potentially generate higher short-circuit current density (*J*_sc_). However, this advantage is accompanied by a relatively lower open-circuit voltage (*V*_oc_ = 0.51 eV), highlighting the intrinsic trade-off between strong light absorption and voltage output. In contrast, Ru-tdt shows the highest *V*_oc_ value (0.92 eV) but the lowest LHE (0.18), suggesting efficient voltage generation but limited photocurrent production. The Ru-dmit and Ru-pdt dyes present a more balanced photovoltaic profile, combining moderate-to-high LHE with favorable *V*_oc_ values. This balance is often regarded as a critical requirement for achieving high overall power conversion efficiency in DSSCs, as it ensures simultaneous enhancement of both current and voltage output. The recombination driving force (Δ*G*_rec_) further differentiates the dyes, with lower Δ*G*_rec_ values indicating reduced electron–hole recombination losses. In this regard, Ru-tdt exhibits the smallest Δ*G*_rec_ (0.98 eV in the gas phase), suggesting a comparatively suppressed recombination pathway. Additionally, the positive and sufficiently large Δ*G*_reg_ values obtained for Ru-dcdmp and Ru-dmit indicate efficient dye regeneration by the iodide/triiodide electrolyte, a crucial factor for sustaining continuous operation and long-term device stability. Taken together, these results suggest that Ru-dcdmp represents a promising sensitizer due to its outstanding light-harvesting ability and favorable electron-injection and regeneration energetics, although its comparatively lower *V*_oc_ indicates the presence of a trade-off between photocurrent generation and voltage output.

**Table 4:** Calculated photovoltaic properties of the Ru-dyes in this investigation (in gas and acetonitrile phases) using the DFT/B3LYP/6-31G(d), LANL2DZ method

Dye	*V*_oc_	*E* ^dye^_ox_	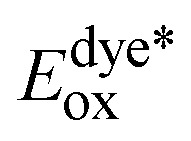	Δ*G*_inj_	Δ*G*_reg_	Δ*G*_rec_	LHE
**Gas phase**							
Ref dye	0.91	5.00	2.39	−1.61	0.20	1.00	0.17
Ru-dcdmp	0.51	5.32	2.75	−1.25	0.52	1.32	0.24
Ru-dmit	0.71	5.22	2.66	−1.34	0.42	1.22	0.21
Ru-pdt	0.84	5.07	2.46	−1.54	0.27	1.07	0.17
Ru-tdt	0.92	4.98	2.39	−1.61	0.18	0.98	0.21

**Acetonitrile phase**							
Ref dye	1.19	5.35	2.73	−1.27	0.55	1.35	0.28
Ru-dcdmp	0.92	5.41	2.71	−1.29	0.61	1.41	0.28
Ru-dmit	1.13	5.40	2.70	−1.30	0.60	1.40	0.24
Ru-pdt	1.14	5.39	2.69	−1.31	0.59	1.39	0.21
Ru-tdt	1.16	5.37	2.80	−1.20	0.57	1.37	0.22

A comparison between gas-phase and acetonitrile solvent results reveals a pronounced solvent effect on the photovoltaic descriptors. In the presence of acetonitrile, the open-circuit voltage increases significantly for all dyes, reflecting improved energetic alignment and reduced charge-recombination tendencies under more realistic operating conditions. For instance, the *V*_oc_ of the reference dye increases from 0.91 eV in the gas phase to 1.19 eV in solution, while Ru-dcdmp and Ru-tdt show corresponding increases from 0.51 to 0.92 eV and from 0.92 to 1.16 eV, respectively. This behavior is consistent with the solvent-induced stabilization and reorganization of the frontier molecular orbital energies, particularly the deeper HOMO levels observed in acetonitrile, which contribute to suppressing interfacial charge recombination and improving the energetic conditions for photovoltage generation.

Overall, the obtained results demonstrate a clear correlation between the solvent-induced electronic reorganization and the optical and photovoltaic responses of the investigated dyes. In acetonitrile, the polar environment modifies the frontier molecular orbital energies, leading to stabilization of the HOMO levels and a relative upward shift of the LUMO energies, thereby increasing the HOMO–LUMO energy gap compared to the gas phase. This electronic reorganization is directly reflected in the optical properties through the pronounced blue shift of the principal MLCT absorption bands. Simultaneously, the oscillator strengths of the dominant electronic transitions increase significantly in solution, indicating enhanced transition probability and more effective electronic coupling associated with intramolecular charge-transfer processes. These solvent-induced effects also contribute to the improved photovoltaic behavior observed in acetonitrile, particularly the substantial enhancement of *V*_oc_ values, which can be attributed to improved energetic alignment and suppressed interfacial charge recombination. Therefore, the combined electronic, optical, and photovoltaic analyses consistently demonstrate that the polar solvent environment plays a crucial role in tuning the performance of Ru-based sensitizers for DSSC applications.

To further elucidate the role of NLO properties, we analyzed the relationship between the computed NLO response and the photovoltaic performance of the dyes. It is important to note that the absolute magnitude of *β* is not a direct, monotonic descriptor of DSSC efficiency, as previously observed in studies investigating the correlation between nonlinear optical properties and charge-transfer efficiency in transition-metal sensitizers.^70^ While the reference dye exhibits a higher *β* value, the superior photovoltaic performance of Ru-dcdmp is primarily attributed to its balanced energy-level alignment and favorable charge-injection kinetics. The enhancement of ICT character in Ru-dcdmp—evidenced by the ∼4.5-fold increase in *β* upon going from the gas phase to acetonitrile—is sufficient to promote efficient electron injection into the TiO_2_ conduction band. In this context, the NLO parameters should be viewed as valuable indicators of electronic redistribution capabilities rather than absolute metrics of power-conversion efficiency. Consistently, the calculated photovoltaic descriptors show improved LHE and thermodynamically favorable electron injection free energy (Δ*G*_inj_ < 0) for all dyes in the solvent phase. Although Δ*G*_inj_ is primarily governed by the alignment of dye excited-state levels with the TiO_2_ conduction band rather than directly by *β*, the increased charge-transfer character implied by larger *β* values promotes more efficient exciton dissociation and interfacial electron transfer at the dye/TiO_2_ interface.

Overall, the combined photovoltaic analysis demonstrates that optimal DSSC sensitizers require a delicate balance among high LHE, sufficiently negative Δ*G*_inj_, appropriate *V*_oc_, minimized Δ*G*_rec_, and favorable Δ*G*_reg_. The present results highlight the effectiveness of dithiolate auxiliary ligand engineering in tuning these interdependent parameters and underline the strong potential of Ru-dcdmp, along with Ru-dmit and Ru-pdt, as efficient sensitizers for next-generation dye-sensitized solar cells.

### 3.5 Dye adsorption and interfacial electronic properties at the TiO_2_ surface

The adsorption of sensitizer dyes onto the semiconductor surface is a critical step in determining the overall efficiency of dye-sensitized solar cells, as it governs both the stability of the device and the efficiency of interfacial electron transfer (IET). In DSSCs, dye attachment typically occurs through anchoring groups such as carboxylic acids, which bind strongly to surface titanium atoms of TiO_2_. Among the possible coordination motifs, the bidentate chelating mode is widely recognized as one of the most favorable adsorption configurations because it generally provides enhanced electronic coupling and improved resistance against dye desorption. Accordingly, bidentate adsorption models were employed for most of the investigated dye–TiO_2_ systems. In contrast, the optimized Ru-dmit@(TiO_2_)_8_ complex adopts a monodentate adsorption mode after full geometry optimization. Frequency calculations confirmed the absence of imaginary frequencies, indicating that the optimized geometry represents a true local minimum on the potential energy surface. Notably, despite its different anchoring configuration, Ru-dmit exhibits the most negative adsorption energy of the investigated series (−2.86 eV), demonstrating that strong dye–surface interactions are preserved even in the monodentate adsorption mode.

To model the dye–semiconductor interface, a (TiO_2_)_8_ nanocluster^71–74^ was employed as a representative fragment of the TiO_2_ surface. This cluster model offers a reasonable compromise between computational efficiency and the description of the local electronic structure and interfacial charge-transfer characteristics. Nevertheless, it should be noted that small TiO_2_ clusters may introduce finite-size effects and do not reproduce the periodic band structure of extended TiO_2_ surfaces, which can influence charge delocalization and band alignment at the interface. More quantitative descriptions of interfacial electronic properties and electron-injection processes generally require larger TiO_2_ clusters or periodic slab models. Despite these limitations, the (TiO_2_)_8_ model remains suitable for examining dye–surface binding configurations and comparing relative adsorption trends among the investigated sensitizers.

To evaluate the thermodynamic stability of the dye@(TiO_2_)_8_ complexes, the adsorption energies (*E*_ads_) were calculated using the (TiO_2_)_8_ cluster model. All geometries were fully optimized at the B3LYP-D3/6-31G(d,p)/LANL2DZ level of theory, ensuring that long-range dispersion effects were consistently accounted for throughout the dye–TiO_2_ interaction. The computed *E*_ads_values, ranging from −2.73 to −2.86 eV, indicate strong and thermodynamically favorable adsorption, confirming the stability of the dye–semiconductor interfaces. It is important to note that, while basis set superposition error (BSSE) correction is a standard refinement in supramolecular chemistry, our methodological approach prioritized the consistency of relative adsorption trends across the dye series. Given the size of the Ru- based dyes, (TiO_2_)_8_ cluster and the objective of our comparative analysis, the BSSE is expected to have a systematic contribution across all systems, thereby preserving the reliability of the observed stability ranking.^75,76^ Consequently, the reported trend serves as a robust indicator of the preferential binding configurations for the investigated ruthenium sensitizers, consistent with prior computational studies in this field.^75^

Full geometry optimizations were performed for all dye@(TiO_2_)_8_ complexes in the gas phase to elucidate the structural stability and electronic features of the adsorbed systems. Key interfacial descriptors, including Ti–O bond lengths, adsorption energies (*E*_ads_), and dipole moments (*µ*), were analyzed to assess the strength and effectiveness of dye adsorption. These parameters are directly linked to interfacial electron injection efficiency and recombination suppression, which are decisive for high photovoltaic performance. The optimized structural parameters and calculated energetic values are summarized in Table 5. The calculated adsorption energies for the investigated complexes lie in the range of −2.73 to −2.86 eV, indicating strong and thermodynamically favorable adsorption of all dyes onto the TiO_2_ surface. Such large negative *E*_ads_ values confirm the formation of stable dye–semiconductor interfaces and suggest good resistance against dye desorption under operational conditions. For most dye–TiO_2_ complexes, the Ti–O bond lengths between the surface titanium atoms and the oxygen atoms of the carboxylate anchoring groups fall within a narrow range of 2.11–2.16 Å, supporting the formation of stable bidentate coordination bonds. In contrast, the Ru-dmit@(TiO_2_)_8_ complex exhibits a monodentate adsorption configuration characterized by a single Ti–O bond to the TiO_2_ surface.

**Table 5:** Optimized structures of dye/(TiO_2_)_8_ complexes and their corresponding parameters, including bond lengths (Ti–O, Å), absorption energies (*E*_ads_), and dipole moments (*µ*)

Dye/(TiO_2_)_8_ structures	
Ref dye@(TiO_2_)_8_	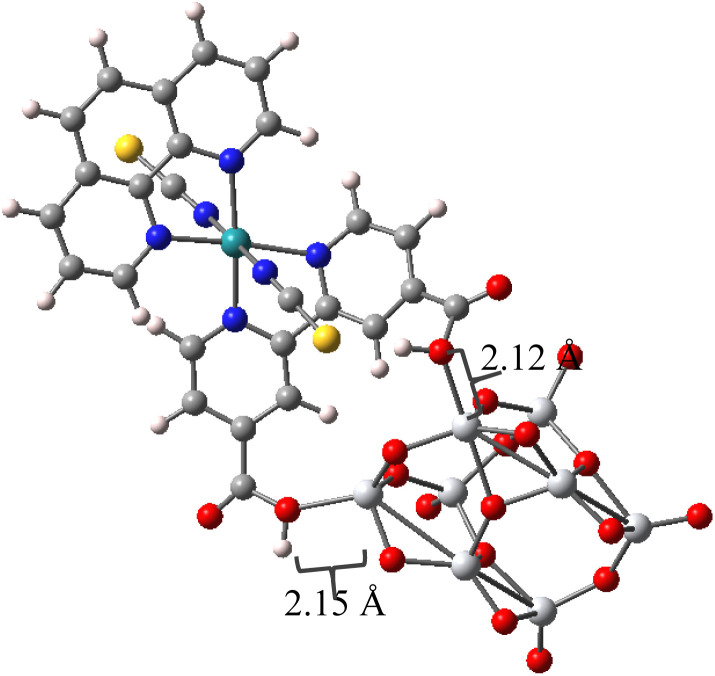
*E*_ads_ = −2.84 eV	
*µ* = 33.05 D	
Ru-dcdmp@(TiO_2_)_8_	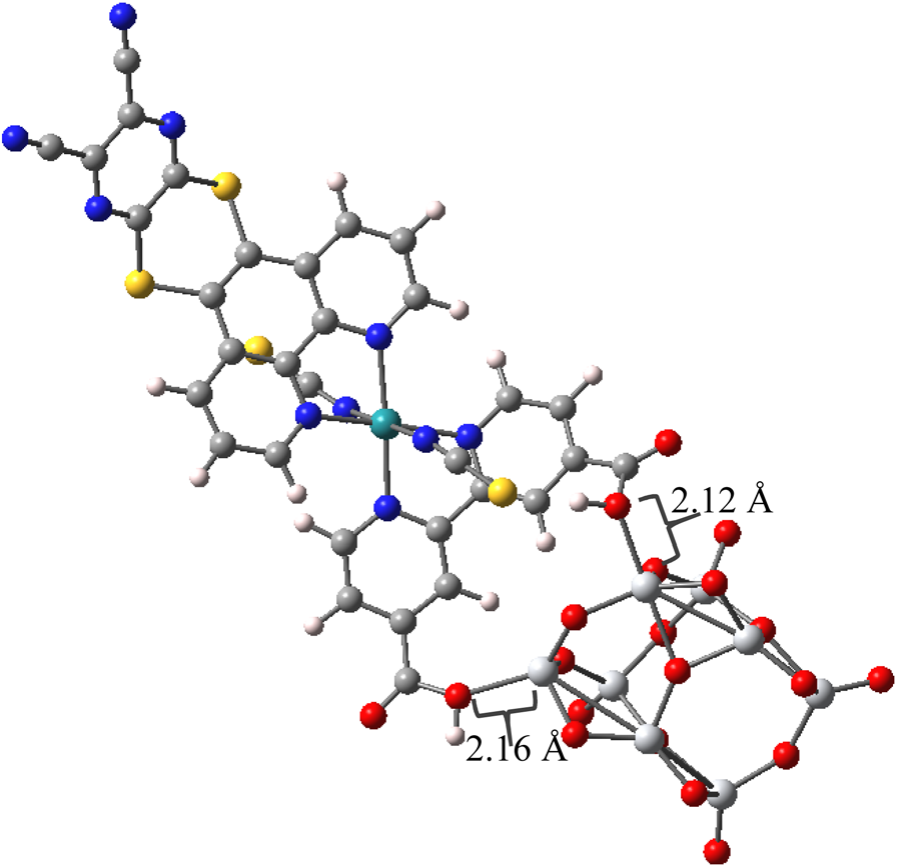
*E*_ads_ = −2.73 eV	
*µ* = 24.80 D	
Ru-dmit@(TiO_2_)_8_	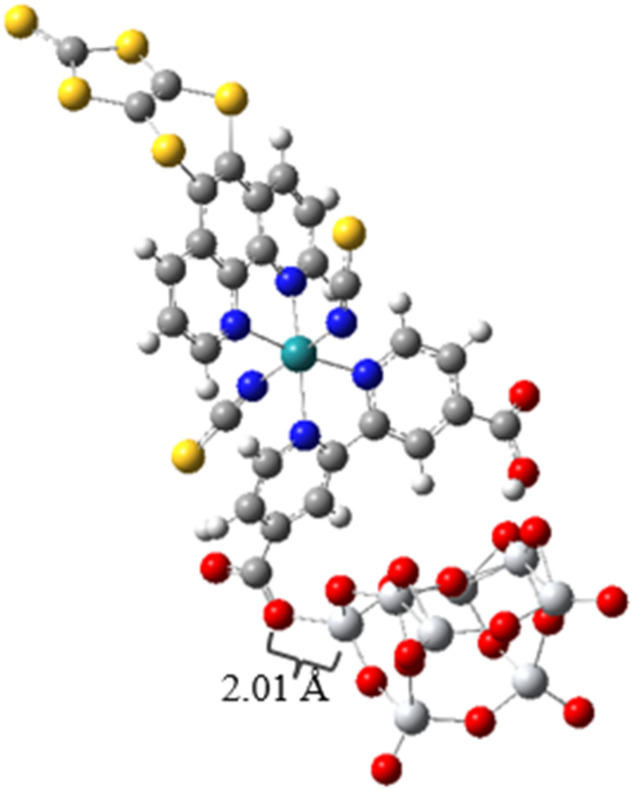
*E*_ads_ = −2.86 eV	
*µ* = 22.75 D	
Ru-pdt@(TiO_2_)_8_	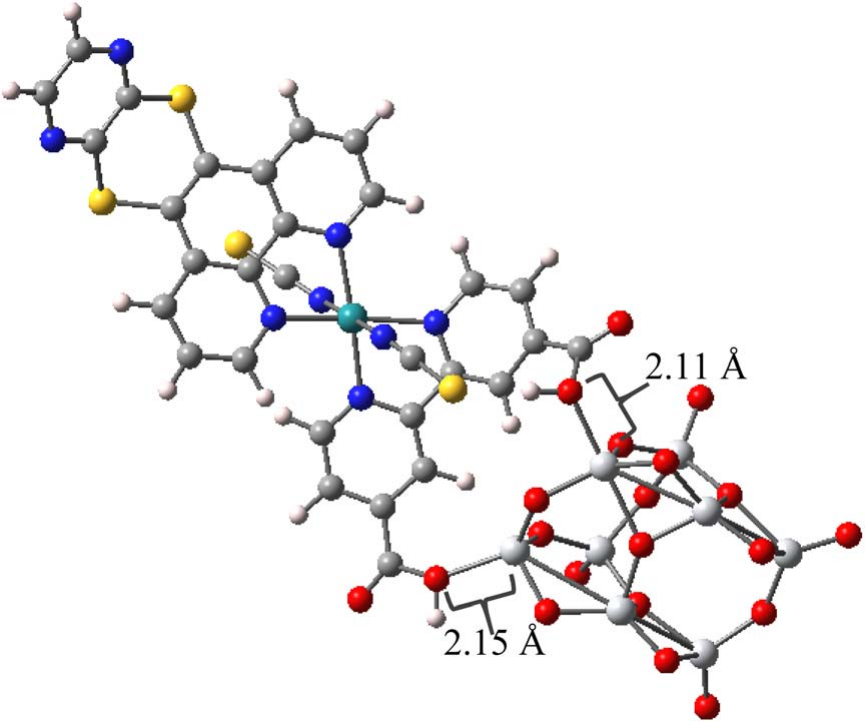
*E*_ads_ = −2.81 eV	
*µ* = 33.53 D	
Ru-tdt@(TiO_2_)_8_	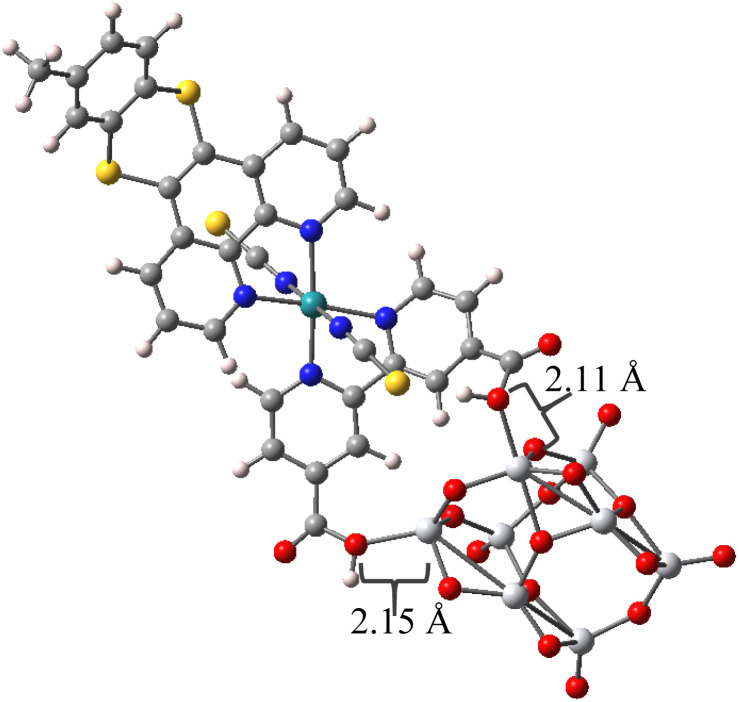
*E*_ads_ = −2.85 eV	
*µ* = 35.63 D	

In addition to structural stability, the interfacial dipole moment plays a crucial role in modulating the electronic alignment at the dye–TiO_2_ interface. The calculated dipole moments of the dye@(TiO_2_)_8_ complexes reveal substantial polarization upon adsorption, reflecting pronounced charge redistribution across the interface. An increased interfacial dipole is known to induce an upward shift of the TiO_2_ conduction band edge, thereby enhancing the driving force for electron injection and potentially increasing the open-circuit voltage. Consequently, the relatively large dipole moments observed for the adsorbed dyes suggest favorable conditions for efficient charge separation and suppressed back-electron transfer. Overall, the combined analysis of adsorption energies, bonding geometries, and interfacial dipole moments demonstrates that all designed ruthenium dyes form strong and electronically favorable interfaces with TiO_2_. These findings confirm that the employed anchoring strategy and auxiliary ligand engineering not only ensure high structural stability but also promote efficient interfacial electron transfer, reinforcing the suitability of these dyes, particularly Ru–dcdmp, for high-performance DSSC applications.

### 3.6. Optical and electronic properties of dyes adsorbed on TiO_2_: interfacial charge-transfer iInsights

**Fig. 5:**
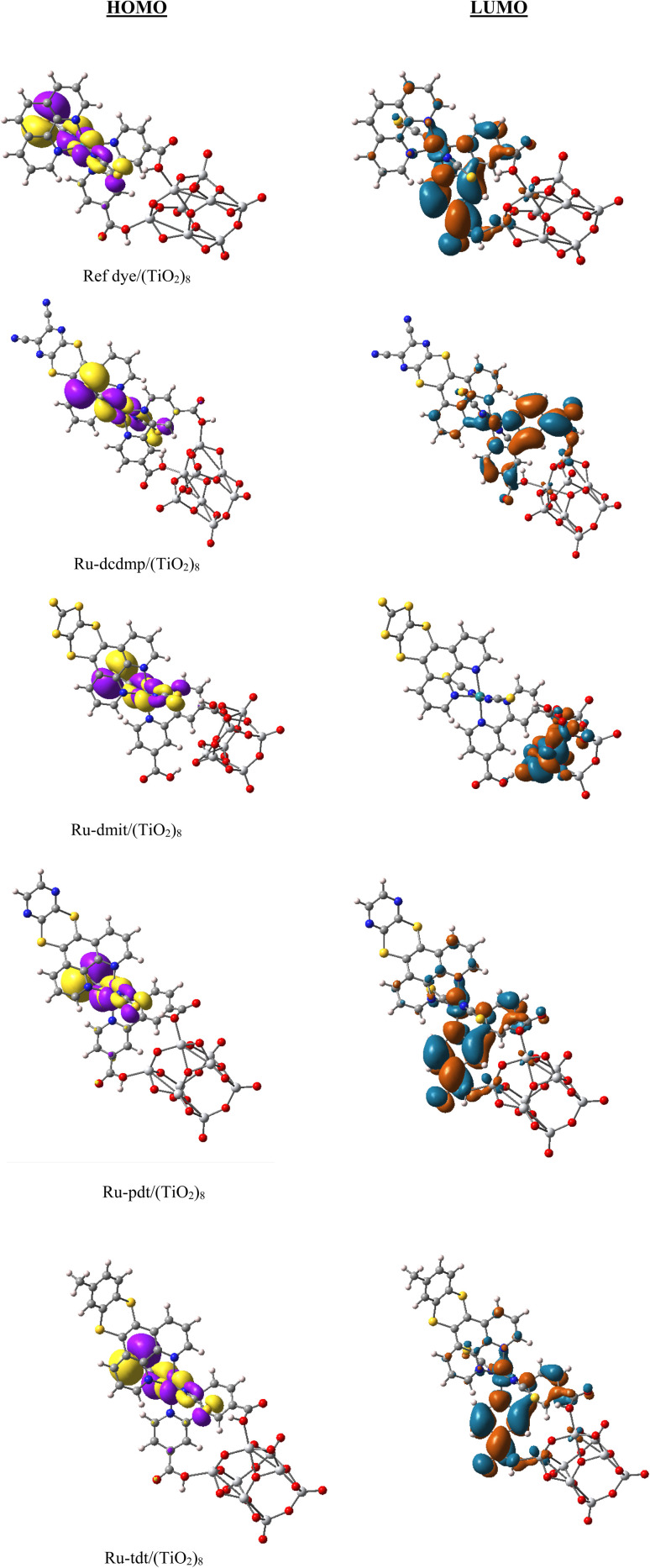
Optimized geometries and frontier molecular orbitals (FMOs) of the dye/(TiO_2_)_8_ complexes in the gas phase.

**Fig. 6:**
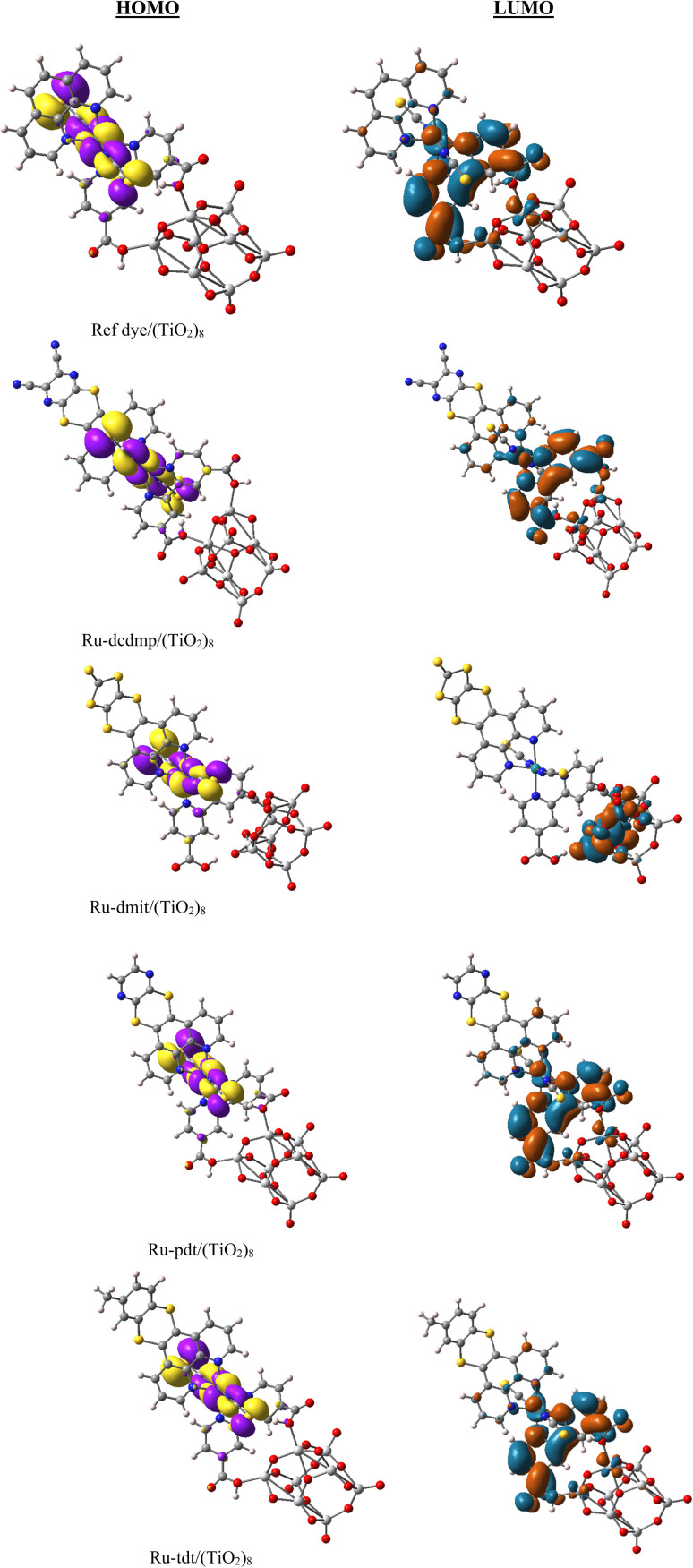
Optimized geometries and frontier molecular orbitals (FMOs) of the dye/(TiO_2_)_8_ complexes in the acetonitrile phase.

The frontier molecular orbitals (FMOs) offer crucial insights into the intramolecular and interfacial charge-transfer characteristics of the dye/(TiO_2_)_8_ systems. The spatial distributions of the HOMO and LUMO levels are depicted in Fig. 5 and 6. For all investigated complexes, the HOMO electron density is predominantly localized on the Ru center with partial delocalization over the thiocyanate ligands, whereas the LUMO is mainly distributed over the anchoring groups and the (TiO_2_)_8_ cluster. This pronounced spatial separation between donor- and acceptor-like orbitals is a clear signature of efficient photoinduced electron injection from the excited dye into the conduction band of TiO_2_, which is a prerequisite for high-performance DSSCs.

**Fig. 7:**
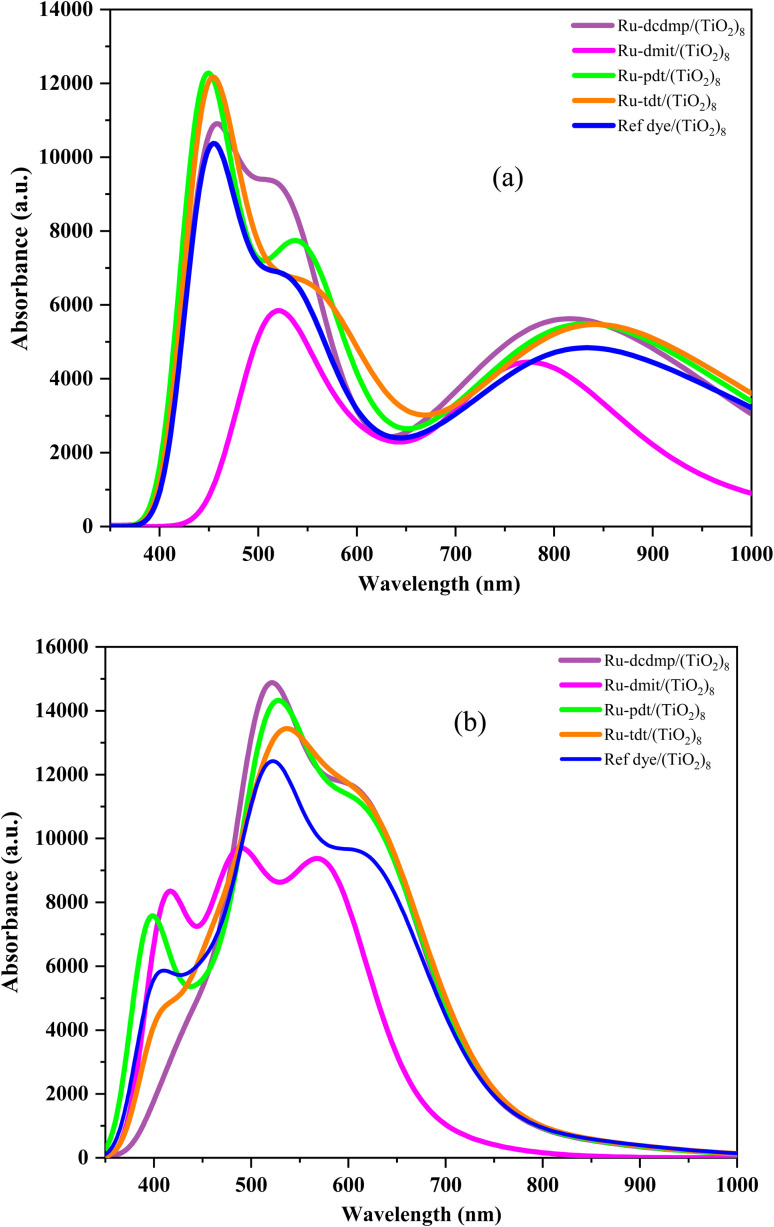
Theoretical UV-visible absorption spectra of dye/(TiO_2_)_8_ complexes calculated at the DFT/B3LYP/6-31G(d), LANL2DZ level in (a) the gas phase and (b) acetonitrile solution.

To further elucidate the optical response and excited-state behavior of the dye–TiO_2_ interfaces, TD-DFT calculations were carried out for the low-lying vertical excitation states of the dye-)TiO_2_(_8_ complexes. The calculated excitation energies (*E*_ex_), absorption maxima (*λ*_max_), oscillator strengths (*f*), light-harvesting efficiencies (LHE), and dominant electronic transitions, obtained at the B3LYP level of theory, are summarized in Fig. 7 and Table 6. The simulated UV-visible absorption spectra were evaluated in both gas and acetonitrile phases to assess the combined effects of semiconductor coupling and solvent polarization.

**Table 6:** The absorption energies (measured in nanometers), oscillator strength (*f*), major transitions (M.T) of the dye/(TiO_2_)_8_ in gas phase and acetonitrile solvent using the DFT/B3LYP/6-31G(d), LANL2DZ method

Dye	State	*E*_ex_	*λ*_max_	*f*	LHE	M.T
**Gas phase**						
Ref Dye/(TiO_2_)_8_	3	1.48	836	0.05	0.10	H−1 → LUMO (11%), HOMO → L+1 (74%)
	32	2.76	449	0.07	0.15	H−8 → LUMO (41%), H−2 → L+4 (18%)
Ru-dcdmp/(TiO_2_)_8_	3	1.54	805	0.05	0.11	H−1 → LUMO (10%), HOMO → L+1 (72%)
	20	2.32	534	0.07	0.15	H−4 → LUMO (46%), H−2 → L+1 (16%)
	39	2.78	447	0.08	0.17	H−9 → LUMO (48%), H−4 → LUMO (14%)
Ru-dmit/(TiO_2_)_8_	5	1.53	812	0.03	0.06	H−1 → L+1 (75%), H−1 → L+2 (16%)
	7	1.63	763	0.02	0.05	H−1 → L+1 (22%), H−1 → L+2 (49%), H−1 → L+3 (12%)
	30	2.39	519	0.03	0.07	H−6 → L+2 (18%), H−4 → L+1 (14%), H−4 → L+2 (10%)
Ru-pdt/(TiO_2_)_8_	3	1.50	829	0.05	0.12	H−1 → LUMO (12%), HOMO → L+1 (73%)
	16	2.26	549	0.05	0.11	H−6 → LUMO (20%), H−4 → LUMO (26%), H−1 → L+3 (26%)
	36	2.81	441	0.07	0.15	H−9 → LUMO (46%), H−6 → LUMO (12%), H−3 → L+2 (10%)
Ru-tdt/(TiO_2_)_8_	3	1.47	842	0.05	0.12	H−1 → LUMO (13%), HOMO → L+1 (72%)
	15	2.18	569	0.04	0.09	H−5 → LUMO (12%), H−3 → LUMO (66%)
	33	2.75	450	0.08	0.16	H−10 → LUMO (26%), H−4 → L+2 (24%), H−2 → L+5 (11%)

**Acetonitrile phase**						
Ref Dye/(TiO_2_)_8_	3	1.97	629	0.07	0.15	H−1 → LUMO (20%), HOMO → L+1 (53%), HOMO → L+2 (14%)
	8	2.39	518	0.13	0.26	H−3 → LUMO (23%), H−2 → LUMO (39%)
Ru-dcdmp/(TiO_2_)_8_	3	2.01	616	0.09	0.20	H−1 → LUMO (18%), HOMO → L+1 (47%), HOMO → L+3 (23%)
	11	2.40	516	0.16	0.30	H−3 → LUMO (14%), H−2 → LUMO (44%)
Ru-dmit/(TiO_2_)_8_	4	2.14	578	0.06	0.12	H−1 → LUMO (17%), H−1 → L+1 (13%), H−1 → L+2 (19%), HOMO → L+1 (31%), HOMO → L+2 (17%)
	14	2.53	491	0.05	0.10	H−3 → L+1 (11%), H−3 → L+2 (21%), H−1 → L+9 (19%), HOMO → L+6 (17%)
	36	3.03	409	0.04	0.08	H−3 → L+1 (21%), H−3 → L+9 (26%), H−2 → L+2 (15%)
Ru-pdt/(TiO_2_)_8_	3	1.99	622	0.09	0.19	H−1 → LUMO (20%), HOMO → L+1 (47%), HOMO → L+2 (21%)
	8	2.38	521	0.15	0.30	H−4 → LUMO (29%), H−2 → LUMO (39%)
	38	3.15	394	0.05	0.11	H−2 → L+8 (13%), H−2 → L+9 (14%), H−1 → L+10 (33%)
Ru-tdt/(TiO_2_)_8_	3	1.98	626	0.09	0.19	H−1 → LUMO (20%), HOMO → L+1 (48%), HOMO → L+2 (18%)
	7	2.33	531	0.12	0.25	H−4 → LUMO (25%), H−2 → LUMO (49%)

Compared to the isolated dyes, all dye–TiO_2_ complexes exhibit a pronounced bathochromic shift upon adsorption, accompanied by a notable enhancement in oscillator strength. In the gas phase, while the isolated dyes absorb predominantly within the 410–795 nm range with moderate oscillator strengths, adsorption onto TiO_2_ induces a substantial red shift of *λ*_max_ to the near-infrared region (≈812–842 nm), along with a reduction in excitation energies to approximately 1.47–1.54 eV. This behavior reflects the stabilization of excited states arising from strong electronic coupling between the dye and the TiO_2_ surface, as well as the emergence of mixed metal-to-ligand and ligand-to-semiconductor charge-transfer character. Analysis of the dominant transitions reveals that, although excitations in isolated dyes are mainly governed by HOMO → LUMO and H−1 → LUMO transitions, dye adsorption significantly increases the contribution of transitions such as HOMO → L+1. This trend highlights the enhanced intermolecular charge-transfer character and stronger orbital hybridization at the dye–TiO_2_ interface, which are highly beneficial for efficient electron injection.

In the acetonitrile phase, solvent effects further modulate the optical properties. Isolated dyes display absorption maxima typically in the 329–585 nm range with relatively higher excitation energies (2.12–3.77 eV). Upon adsorption onto TiO_2_, the absorption bands shift toward longer wavelengths (≈394–629 nm), accompanied by a marked decrease in excitation energies (≈1.97–3.15 eV) and a noticeable increase in oscillator strength (up to ∼0.16). Consequently, the calculated LHE values are significantly enhanced for the dye–TiO_2_ systems, indicating more efficient photon harvesting and improved charge-transfer capability under realistic device-relevant conditions.

Overall, these results demonstrate that dye adsorption onto TiO_2_ not only broadens the spectral response toward the visible and near-infrared regions but also strengthens interfacial electronic coupling and charge-transfer efficiency. Such combined optical and electronic enhancements strongly support the suitability of the designed dyes as effective sensitizers for high-performance DSSC applications.

## 4 Conclusion

In this work, the impact of dithiolate auxiliary ligands on the electronic, optical, and photovoltaic properties of ruthenium-based sensitizers for dye-sensitized solar cells (DSSCs) was systematically explored using density functional theory (DFT) and time-dependent DFT (TD-DFT) calculations. Four newly designed complexes, Ru-dcdmp, Ru-dmit, Ru-pdt, and Ru-tdt, were investigated and benchmarked against a reference dye, with a particular focus on ligand-induced modulation of light absorption, charge-transfer characteristics, and dye–TiO_2_ interfacial behavior. The results demonstrate that incorporating dithiolate ligands effectively tailors the electronic structure of the dyes by narrowing the HOMO–LUMO energy gaps and enhancing intramolecular charge transfer (ICT). All designed complexes exhibit smaller energy gaps than the reference dye, facilitating photoexcitation and electron delocalization. Among them, Ru-dcdmp shows the most favorable electronic structure, characterized by the smallest band gap and an optimal energetic alignment with the TiO_2_ conduction band, ensuring a spontaneous and efficient electron injection process.

TD-DFT simulations reveal that ligand engineering significantly improves the absorption profiles of the dyes, leading to broadened and intensified absorption across the visible and near-infrared regions. Solvent effects further enhance these properties, inducing spectral shifts and increased oscillator strengths in acetonitrile, which are highly desirable under realistic operating conditions. Although the long-wavelength MLCT transitions remain relatively invariant across the complexes, the auxiliary ligands markedly influence absorption intensities and higher-energy transitions, thereby improving overall light-harvesting efficiency. Photovoltaic analyses indicate that Ru-dcdmp exhibits the most balanced and promising performance, combining the highest light-harvesting efficiency with a strong driving force for electron injection and efficient dye regeneration. Adsorption studies on a (TiO_2_)_8_ cluster confirm robust dye–semiconductor interactions. Most complexes exhibit stable bidentate anchoring to the TiO_2_ surface, whereas the Ru-dmit complex adopts a monodentate adsorption mode while still displaying the strongest adsorption energy of the series (−2.86 eV), indicating strong interfacial stabilization despite the different anchoring configuration. The spatial distribution of frontier molecular orbitals, HOMO localized on the Ru center and NCS ligands and LUMO delocalized over the anchoring group and TiO_2_, clearly supports efficient interfacial charge transfer, a key requirement for high DSSC efficiency.

In conclusion, this study highlights dithiolate ligands, particularly dcdmp, as powerful molecular design elements for optimizing ruthenium-based sensitizers. By simultaneously enhancing electronic structure, optical absorption, and interfacial charge-transfer properties, these ligands enable the rational design of high-performance dyes with extended spectral response and improved photovoltaic characteristics. The insights gained from this computational investigation provide a solid theoretical foundation for the experimental synthesis and device-level evaluation of next-generation DSSCs with enhanced power conversion efficiency.

## Conflicts of interest

There are no conflicts to declare.

## Supplementary Material

RA-016-D6RA01663D-s001

## Data Availability

All data supporting this study are included in the article and its supplementary information (SI). Supplementary information: additional tables, optimized structural coordinates, and other relevant computational data supporting the findings of this study See DOI: https://doi.org/10.1039/d6ra01663d.

## References

[cit1] Abdellah I. M. (2025). RSC Adv..

[cit2] Maoz M., Abbas Z., Shah S. A. B., Lughi V. (2025). Sustainability.

[cit3] Mariotti N., Bonomo M., Fagiolari L., Barbero N., Gerbaldi C., Bella F., Barolo C. (2020). Green Chem..

[cit4] Malashi N. M., Jande Y. A. C., Wazzan N., Safi Z., Al-Qurashi O. S., Costa R. (2024). J. Mol. Graphics Modell..

[cit5] Shamsuddin M. A., Hassan N. I., Su’ait M. S., Ibrahim S. (2026). Sol. Energy.

[cit6] Xin X., He M., Han W., Jung J., Lin Z. (2011). Angew. Chem..

[cit7] O’regan B., Grätzel M. (1991). Nature.

[cit8] Hagfeldt A., Boschloo G., Sun L., Kloo L., Pettersson H. (2010). Chem. Rev..

[cit9] Malhotra S. S., Ahmed M., Gupta M. K., Ansari A. (2024). Sustain. Energy Fuels.

[cit10] Muliani L. , Hidayat J. and Anggraini P. N., in AIP Conference Proceedings, AIP Publishing, 2016, vol. 1725

[cit11] Joy E. J., Mathew E., Mathew M., Thomas A. P. (2025). Sustain. Chem. Clim. Action.

[cit12] Al-Alwani M. A. M., Mohamad A. B., Ludin N. A., Kadhum A. A. H., Sopian K. (2016). Renew. Sustain. Energy Rev..

[cit13] Portillo-Cortez K., Martínez A. (2022). Comput. Theor. Chem..

[cit14] Mahalakshmi S. (2025). Coord. Chem. Rev..

[cit15] Sen A., Groß A. (2019). Int. J. Quantum Chem..

[cit16] Dong R., Calzolari A., di Felice R., El-Shafei A., Hussain M., Buongiorno Nardelli M. (2014). Journal of Physical Chemistry.

[cit17] Karges J., Heinemann F., Jakubaszek M., Maschietto F., Subecz C., Dotou M., Vinck R., Blacque O., Tharaud M., Goud B. (2020). J. Am. Chem. Soc..

[cit18] Adeloye A. O., Ajibade P. A. (2014). Molecules.

[cit19] Aghazada S., Nazeeruddin M. K. (2018). Inorganics.

[cit20] Wu W., Li Y., Zhang J., Guo X., Wang L., Ågren H. (2024). Sol. Energy.

[cit21] Li Y.-L., Li A.-J., Huang S.-L., Vittal J. J., Yang G.-Y. (2023). Chem. Soc. Rev..

[cit22] Al-horaibi S. A., Al-Odayni A.-B., Alezzy A., ALSaeedy M., Saeed W., Hasan A., El-Shishtawy R. M. (2023). J. Mol. Struct..

[cit23] Linfoot C. L., Richardson P., McCall K. L., Durrant J. R., Morandeira A., Robertson N. (2011). Sol. Energy.

[cit24] May A. M., Dempsey J. L. (2024). Chem. Sci..

[cit25] Alolyan R. A., Wazzan N. (2025). J. Mol. Graphics Modell..

[cit26] Zarate X., Schott-Verdugo S., Rodriguez-Serrano A., Schott E. (2016). J. Phys. Chem. A.

[cit27] Schott E., Zarate X., Arratia-Perez R. (2013). Dyes Pigm..

[cit28] Cheshire T. P., Houle F. A. (2021). J. Phys. Chem. A.

[cit29] Talbot J. J., Cheshire T. P., Cotton S. J., Houle F. A., Head-Gordon M. (2024). J. Phys. Chem. A.

[cit30] Mitsopoulou C. A. (2010). Coord. Chem. Rev..

[cit31] Kerraj S., Salah M., Chtita S., El Idrissi M., Belaaouad S., Mohammed M., Acharjee N., Komiha N. (2022). Comput. Theor. Chem..

[cit32] Samiee S., Taghvaeian S. (2018). Spectrochim. Acta A Mol. Biomol. Spectrosc..

[cit33] ea Frisch M. J. , Trucks G. W., Schlegel H. B., Scuseria G. E., Robb Ma., Cheeseman J. R., Scalmani G., Barone V., Petersson G. A. and Nakatsuji H., Gaussian, Inc., Wallingford, CT, 2016

[cit34] Becke A. D. (1993). J. Phys. Chem..

[cit35] Lee C., Yang W., Parr R. G. (1988). Phys. Rev. B:Condens. Matter Mater. Phys..

[cit36] Grimme S., Antony J., Ehrlich S., Krieg H. (2010). J. Phys. Chem..

[cit37] Hay P. J., Wadt W. R. (1985). J. Phys. Chem..

[cit38] Dunning Jr T. H. and Hay P. J., in Methods of electronic structure theory, Springer, 1977, pp. 1–27

[cit39] McWeeny R. (1950). Nature.

[cit40] Ditchfield R., Hehre W. J., Pople J. A. (1971). J. Phys. Chem..

[cit41] Foresman J. and Frish E., Gaussian Inc., Pittsburg, USA, 1996, 21, 93–123

[cit42] Hay P. J., Wadt W. R. (1985). J. Phys. Chem..

[cit43] Mennucci B., Tomasi J. (1997). J. Phys. Chem..

[cit44] Matovic L., Tasic N., Trisovic N., Ladarevic J., Vitnik V., Vitnik Z., Grgur B., Mijin D. (2019). Turk. J. Chem..

[cit45] Parr R. G., Pearson R. G. (1983). J. Am. Chem. Soc..

[cit46] Lu T., Chen F. (2012). J. Comput. Chem..

[cit47] El Karkri A., El Malki Z., Bouachrine M., Serein-Spirau F., Sotiropoulos J.-M. (2020). RSC Adv..

[cit48] Zhang J., Li H.-B., Sun S.-L., Geng Y., Wu Y., Su Z.-M. (2012). J. Mater. Chem..

[cit49] Fan W., Tan D., Deng W. (2012). ChemPhysChem.

[cit50] Xu J., Zhu L., Fang D., Chen B., Liu L., Wang L., Xu W. (2012). ChemPhysChem.

[cit51] Sang-aroon W., Saekow S., Amornkitbamrung V. (2012). J. Photochem. Photobiol., A.

[cit52] Daeneke T., Mozer A. J., Uemura Y., Makuta S., Fekete M., Tachibana Y., Koumura N., Bach U., Spiccia L. (2012). J. Am. Chem. Soc..

[cit53] Glendening E. D., Landis C. R., Weinhold F. (2012). Wiley Interdiscip. Rev.: Comput. Mol. Sci..

[cit54] Fan W., Tan D., Deng W. (2012). ChemPhysChem.

[cit55] Xu J., Zhu L., Fang D., Chen B., Liu L., Wang L., Xu W. (2012). ChemPhysChem.

[cit56] Sang-aroon W., Saekow S., Amornkitbamrung V. (2012). J. Photochem. Photobiol., A.

[cit57] Patil D. S., Avhad K. C., Sekar N. (2018). Comput. Theor. Chem..

[cit58] Cherifi K., Cheknane A., Benghia A., Hilal H. S., Rahmoun K., Benyoucef B., Goumri-Said S. (2019). Mater. Today Energy.

[cit59] Fantacci S., De Angelis F., Selloni A. (2003). J. Am. Chem. Soc..

[cit60] Hagfeldt A., Boschloo G., Sun L., Kloo L., Pettersson H. (2010). Chem. Rev..

[cit61] Rana P. J. S., Singh P., Kar P. (2017). ChemistrySelect.

[cit62] Moore B., Sun H., Govind N., Kowalski K., Autschbach J. (2015). J. Chem. Theory Comput..

[cit63] Begam K., Bhandari S., Maiti B., Dunietz B. D. (2020). J. Chem. Theory Comput..

[cit64] Atkins A. J., Talotta F., Freitag L., Boggio-Pasqua M., González L. (2017). J. Chem. Theory Comput..

[cit65] Hu K., Severin H. A., Koivisto B. D., Robson K. C. D., Schott E., Arratia-Perez R., Meyer G. J., Berlinguette C. P. (2014). Journal of Physical Chemistry.

[cit66] Qu Z., Kroes G.-J. (2006). J. Phys. Chem. B.

[cit67] Fang H., Ma J., Wilhelm M. J., DeLacy B. G., Dai H.-L. (2021). Part. Part. Syst. Charact..

[cit68] Jr L. K. K., Dupont N., Ray L., Ding J., Kovnir K., Hoyt J. M., Hauser A., Shatruk M. (2013). Inorg. Chem..

[cit69] Hua S.-A., Cattaneo M., Oelschlegel M., Heindl M., Schmid L., Dechert S., Wenger O. S., Siewert I., González L., Meyer F. (2020). Inorg. Chem..

[cit70] Sharma S. J., Sekar N. (2024). Int. J. Quantum Chem..

[cit71] Steinke S. J., Piechota E. J., Loftus L. M., Turro C. (2022). J. Am. Chem. Soc..

[cit72] Syzgantseva O. A., Gonzalez-Navarrete P., Calatayud M., Bromley S., Minot C. (2011). J. Phys. Chem..

[cit73] Souilah M., Hachi M., Fitri A., Benjelloun A. T., Benzakour M., Mcharfi M., Zgou H. (2023). Chem. Data Collect..

[cit74] Paredes-Gil K., Mendizabal F., Páez-Hernández D., Arratia-Pérez R. (2017). Comput. Mater. Sci..

[cit75] Nazeeruddin M. K., De Angelis F., Fantacci S., Selloni A., Viscardi G., Liska P., Ito S., Takeru B., Grätzel M. (2005). J. Am. Chem. Soc..

[cit76] Chen Y., Zhang Y., Shen Y., Zhao Y., Qiu Y.-Q. (2022). Phys. Chem. Chem. Phys..

