# Supplementary material for: Dithiolate auxiliary ligands as electronic modulators in ruthenium-based sensitizers: a DFT and TD-DFT study
Source: RSC Adv. 2026 Jul 2;16(34):32566–84. doi: 10.1039/d6ra01663d (PMC13325799; doi:10.1039/d6ra01663d)
Supplement: RA-016-D6RA01663D-s001 [file RA-016-D6RA01663D-s001.pdf]

Supplementary Information (SI) for RSC Advances.
This journal is © The Royal Society of Chemistry 2026

Supplementary Information for:

Dithiolate Auxiliary Ligands as Electronic Modulators in Ruthenium-Based Sensitizers: A DFT and TD-DFT Study

Zohreh Abdollahi, Sepideh Samiee, Zabiollah Mahdavifar*

Department of Chemistry, Faculty of Science, Shahid Chamran University of Ahvaz, Ahvaz, Iran

ORCID: 0000-0002-9709-0502 (Zabiollah Mahdavifar)

*To whom correspondence should be addressed

Fax: ++98-611-3331042

E-mail: z_mahdavifar@scu.ac.ir

Contentes

Table S1. Comparison of calculated structural parameters for the optimized Ru based dyes, including selected bond lengths (Å) and bond angles (°), obtained at the DFT/B3LYP/6 31G(d)/LANL2DZ level in the gas phase and acetonitrile, together with available experimental data; atom numbering corresponds to Fig. 1……………………………………………………3

Table S2. Calculated maximum absorption wavelengths (λmax) and band-gap energies of the N3 dye in ethanol using different density functionals, together with comparison to available experimental data.. 4

Table S3. Calculated bond lengths (Å) of the optimized N3 dye obtained using the B3LYP, CAM-B3LYP, M06-2X, ωB97XD, and PBEPBE functionals in ethanol, together with comparison to available experimental solid-state data for the N3 dye. 4

Fig. SI 1. Optimized structures and frontier molecular orbitals (FMOs) of the Ru dyes calculated at the DFT/B3LYP/6-31G(d), LANL2DZ level in acetonitrile phase. 5

Relaxed Cartesian coordinates of structures 6

Table S1. Comparison of calculated structural parameters for the optimized Ru-based dyes, including selected bond lengths (Å) and bond angles (°), obtained at the DFT/B3LYP/6-31G(d)/LANL2DZ level in the gas phase and acetonitrile, together with available experimental data; atom numbering corresponds to Fig. 1.

	Ref Dye	Ru-dcdmp	Ru-dmit	Ru-pdt	Ru-tdt	Exp.[56]	Exp.[53]
**Gas phase**							
Ru-N1	2.14	2.13	2.13	2.13	2.13	2.06	2.05
Ru-N2	2.08	2.09	2.09	2.09	2.09	2.03	2.04
Ru-N3	2.06	2.07	2.06	2.06	2.06	2.06	2.05
Ru-N4	2.14	2.14	2.14	2.14	2.14	2.03	-
Ru-N5	2.09	2.09	2.09	2.09	2.09	2.05	-
Ru-N6	2.07	2.07	2.07	2.07	2.07	2.05	-
N5=C1	1.18	1.18	1.18	1.18	1.18	-	1.12
C1=S1	1.64	1.64	1.64	1.64	1.64	-	1.65
N1-Ru-N2	77.87	77.42	77.45	77.43	77.47	79.80	78.70
N1-Ru-N5	94.70	94.17	94.15	94.05	94.09	97.80	97.20
N2-Ru-N5	93.26	94.24	94.09	94.04	93.82	95.90	96.40
N2-Ru-N4	173.41	174.88	174.64	174.53	174.23	174.50	173.00
**Acetonitrile phase**							
Ru-N1	2.15	2.13	2.13	2.13	2.13	2.06	2.05
Ru-N2	2.08	2.09	2.09	2.09	2.09	2.03	2.04
Ru-N3	2.06	2.06	2.07	2.06	2.07	2.06	2.05
Ru-N4	2.14	2.13	2.14	2.14	2.13	2.03	-
Ru-N5	2.09	2.09	2.08	2.09	2.08	2.05	-
Ru-N6	2.07	2.07	2.07	2.08	2.07	2.05	-
N5=C1	1.18	1.18	1.18	1.18	1.18	-	1.12
C1=S1	1.64	1.64	1.64	1.64	1.64	-	1.65
N1-Ru-N2	77.87	77.42	77.44	77.43	77.46	79.80	78.70
N1-Ru-N5	94.70	94.17	94.15	94.05	94.09	97.80	97.20
N2-Ru-N5	93.26	94.24	94.09	94.04	93.82	95.90	96.40
N2-Ru-N4	173.41	174.88	174.64	174.53	174.23	174.50	173.00
N3-Ru-N6	90.66	91.34	91.28	91.20	91.11	90.60	90.70

Table S2. Calculated maximum absorption wavelengths (λ_{max}), band gap energies, oscillator strength (*f*) and light-harvesting efficiency (LHE) using B3LYP, CAM-B3LYP, M062x, WB97XD, PBEIPBE functionals and compare with experimental data for N3 dye in ethanol phase.

Functional	B3LYP	CAM-B3LYP	M062X	WB97XD	PBEPBE	Exp[a]
Band gap(eV)	1.76	5.03	4.97	6.13	0.89	1.68
λ_{max} (nm)	393	269	221	271	472	385
f	0.34	0.31	0.24	0.41	0.11	-
LHE	0.54	0.51	0.42	0.61	0.21	-

[a]Exprimental data from Ref [1]

Table S3. Calculated bond lengths (Å) of the optimized N3 dye obtained using the B3LYP, CAM-B3LYP, M06-2X, ωB97XD, and PBEPBE functionals in ethanol, together with comparison to available experimental solid-state data for the N3 dye.

	B3LYP	CAM-B3LYP	M062X	WB97XD	PBEPBE	Exp[b]
Ru-N1	2.04	2.11	2.15	2.16	2.08	2.03
Ru-N2	2.04	2.07	2.09	2.08	2.03	2.03
Ru-N3	2.06	2.10	2.11	2.06	2.06	2.05
Ru-N4	2.09	2.09	2.22	2.10	2.12	2.04
Ru-N5	2.06	2.18	2.15	2.08	2.01	2.04
Ru-N6	2.07	2.15	2.10	2.09	2.06	2.04

[a]Exprimental data from Ref [1]

Reference:

1- K. Cherifi, A. Cheknane, A. Benghia, H. S. Hilal, K. Rahmoun, B. Benyoucef and S. Goumri-Said, *Mater. Today Energy*, 2019, **13**, 109–118.

Dye	HOMO	LUMO

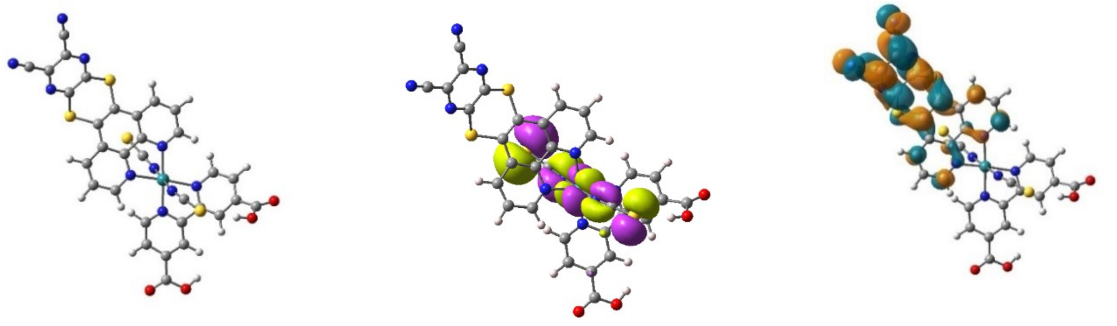

Ru-dcdmp

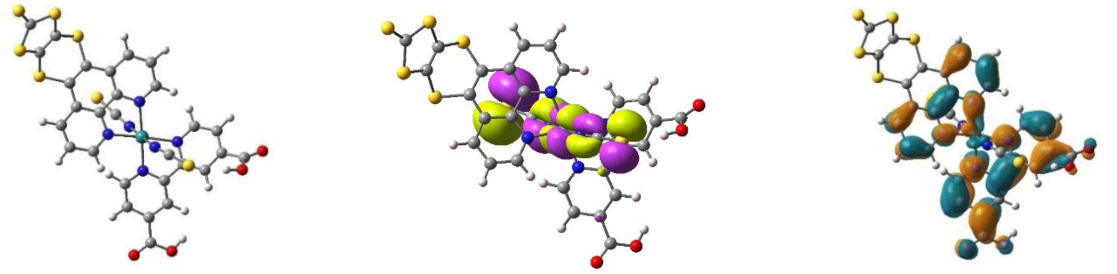

Ru-dmit

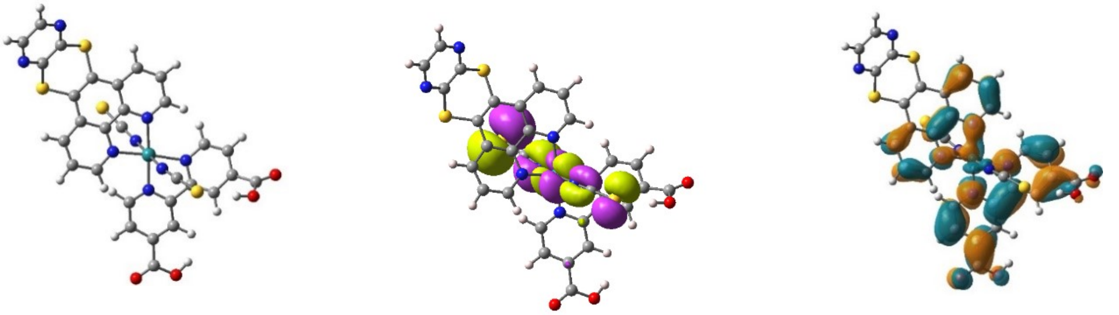

Ru-pdt

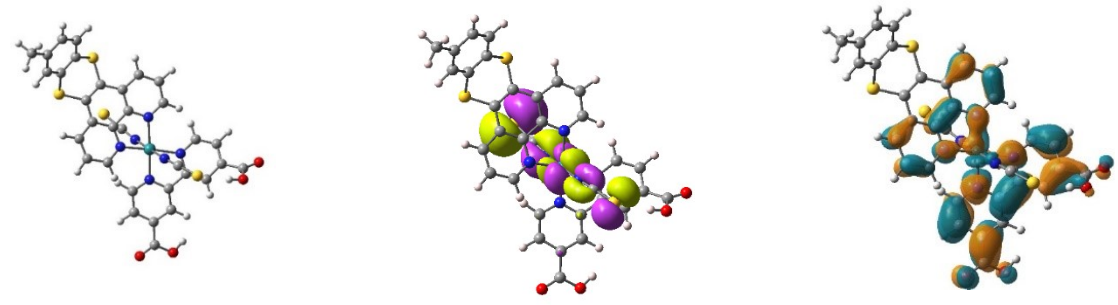

Ru-tdt

Fig. SI 1. Optimized structures and frontier molecular orbitals (FMOs) of the Ru dyes calculated at the DFT/B3LYP/6-31G(d), LANL2DZ level in acetonitrile phase.

Relaxed Cartesian coordinates of structures

Cartesian coordinates of **$(TiO_2)_8$** -optimized structure by B3LYP / 6-31G(d,p);LanL2DZ level of theory
Charge = 0 Multiplicity = 1

Ti	0.28808500	-1.68822500	1.96498700
Ti	-1.37919500	1.22594000	1.91060000
Ti	-1.50058600	-2.12220200	-0.86716100
Ti	-3.56896500	0.33555900	-0.76392500
O	-0.13475600	-3.00770200	-1.65417700
O	-2.53134400	1.37484800	-1.75798300
O	-0.33917400	-0.09582600	2.61250800
O	-1.77208800	2.38330100	2.97355800
O	-1.21479200	-2.27749300	0.80981700
O	-2.94844500	0.57308600	0.81837500
O	0.67571800	-2.75408600	3.12040600
O	-3.18290700	-1.33255300	-1.25404200
Ti	3.30268700	-0.29735100	-0.11959300
Ti	1.52122900	2.66040000	-0.27016500
Ti	1.19027400	-1.73028500	-1.22850600
Ti	-0.69439600	1.31662900	-1.31458600
O	2.63307100	-1.05368500	-1.86770900
O	0.60242900	2.18836700	-2.00320200
O	2.86332000	1.46862900	0.09343100
O	1.84093400	4.22422400	-0.00866200
O	1.69419700	-1.46915700	0.49286400
O	-0.29781600	1.90411800	0.35481600
O	4.77094400	-0.75044000	0.38560500
O	-0.34690700	-0.55190900	-1.22264600

Cartesian coordinates of DFT-optimized structure of **Ref Dye** by B3LYP / 6-31G(d,p);LanL2DZ level of theory
Charge = 0 Multiplicity = 1

C	-2.31005400	-0.43371800	-0.62321300
C	-1.22615500	-2.26291600	-1.55233100
C	-2.19604800	1.00897300	-0.36605100
C	-3.45864900	-1.18478300	-0.39597900
C	-2.36026900	-3.05850300	-1.42081800
H	-0.31832500	-2.63982300	-2.00701000
C	-3.28113000	1.88649100	-0.35525100
C	-3.45906700	-2.53862800	-0.73443900
H	-2.36952500	-4.08426200	-1.77192500
C	-3.05294200	3.26240900	-0.28495700
C	-0.69576800	2.79096700	-0.26847800
C	-1.72805800	3.71645800	-0.25773100
H	0.34420900	3.09245200	-0.28957500
H	-1.52425400	4.78054300	-0.23110000
N	-1.16209800	-1.00998600	-1.07964600

N	-0.91964900	1.46298100	-0.26448500
H	-4.28658100	1.48021900	-0.39733800
H	-4.31202900	-0.74465500	0.10686400
C	-4.59396900	-3.43997800	-0.33172700
C	-4.15137500	4.28808300	-0.22737400
O	-5.07949900	-4.25133600	-1.08444600
O	-3.97572600	5.39082300	0.22985500
O	-5.36660900	3.92318800	-0.70589900
O	-5.02326900	-3.26826300	0.93064500
H	-4.36262500	-2.76926900	1.47098000
H	-5.31493400	3.08867900	-1.19944600
N	1.46500600	0.69333300	-1.79490100
N	-0.49131400	-0.77040800	1.57208700
C	2.49458300	0.98662700	-2.30935300
C	-1.39440000	-1.23915200	2.17333400
S	-2.66817200	-1.87694800	2.99184300
S	3.90336400	1.38721100	-3.01711100
Ru	0.49378800	-0.03335500	-0.12335900
C	2.97998800	2.70434700	2.26076200
C	4.24343800	2.17898900	2.06225100
C	4.37881900	0.95516300	1.37270800
C	3.19874800	0.35430300	0.87971100
C	1.86261600	2.04745600	1.71886700
C	5.63005600	0.27876000	1.17677400
C	3.24482000	-0.94627400	0.28774200
C	4.48086300	-1.62180000	0.17066200
C	5.67869400	-0.95399000	0.59675100
C	4.45411200	-2.94025000	-0.33106900
H	5.38217900	-3.49193000	-0.45050700
C	3.23675100	-3.51719900	-0.63741400
C	2.05776800	-2.76639100	-0.48888500
H	2.83341400	3.62167100	2.82073000
H	5.12546900	2.68074500	2.44962500
H	0.86113300	2.43469800	1.86198900
H	3.17144300	-4.53883900	-0.99588700
H	1.09362500	-3.20400000	-0.70930400
N	2.05153400	-1.50224200	-0.07397200
N	1.96794800	0.92770300	1.00389300
H	6.53946900	0.76466200	1.51797000
H	6.62819600	-1.46511400	0.46698100

Cartesian coordinates of DFT-optimized structure of **Ru-dcdmp** by B3LYP / 6-31G(d,p);LanL2DZ level of theory
Charge = 0 Multiplicity = 1

C	4.47079700	-0.60418700	0.29604900
C	3.39871900	-2.35482100	1.38128600
C	4.43791000	0.84796000	0.06843900
C	5.49846500	-1.44436300	-0.11926000
C	4.42517400	-3.23914300	1.06310400
H	2.54665900	-2.66252100	1.97550300

C	5.57528200	1.63116400	-0.13335000
C	5.43753600	-2.79960300	0.20836600
H	4.40495500	-4.26819700	1.40398600
C	5.45594500	3.02189500	-0.16779100
C	3.10005700	2.75089500	0.22291000
C	4.18949300	3.58649200	0.02914200
H	2.10785900	3.14039400	0.41460600
H	4.07487100	4.66416800	0.02958700
N	3.36807200	-1.09283400	0.92972400
N	3.20720700	1.40931400	0.19268300
H	6.53465100	1.14174700	-0.26654500
H	6.29423600	-1.06832600	-0.75184200
C	6.41102900	-3.78471200	-0.37940600
C	6.61101100	3.95328700	-0.41924800
O	6.94298900	-4.64586800	0.28006200
O	6.44993700	5.07002600	-0.84613800
O	7.85506600	3.48469900	-0.15578400
O	6.63893200	-3.62702400	-1.69501200
H	5.94433100	-3.06473700	-2.11545900
H	7.82206600	2.65370200	0.34524400
N	1.06525900	0.76490700	2.13558900
N	2.27354000	-0.72580900	-1.56482600
C	0.11911900	1.06163900	2.79101900
C	3.01832900	-1.27026600	-2.30465900
S	4.07070500	-2.01470200	-3.32053500
S	-1.19192100	1.46043000	3.66734200
Ru	1.66784700	0.03403400	0.28887400
C	-1.00325900	3.01690100	-1.51288600
C	-2.23389600	2.61558500	-1.02562600
C	-2.33015800	1.40597600	-0.30083300
C	-1.13493600	0.67953700	-0.10600700
C	0.13977900	2.25604100	-1.22935300
C	-3.55350300	0.86052700	0.25060800
C	-1.16597100	-0.64323500	0.43669700
C	-2.39552800	-1.23439100	0.80311300
C	-3.58362800	-0.40533600	0.78413600
C	-2.36836700	-2.60248100	1.15638400
H	-3.28193900	-3.11650500	1.43192500
C	-1.16926400	-3.28817900	1.10979200
C	0.00851000	-2.60703400	0.76851800
H	-0.90758800	3.91928100	-2.10677200
H	-3.11952500	3.20808900	-1.22395400
H	1.11893100	2.54909200	-1.58852300
H	-1.12437500	-4.34732300	1.33847800
H	0.95907700	-3.12163300	0.72195900
N	0.02490500	-1.30872800	0.47720200
N	0.08547900	1.13792100	-0.50633100
S	-4.99336000	1.92251400	0.22333000
S	-5.06610600	-1.10421700	1.50279400
C	-6.30234800	-0.54270200	0.36252000
C	-6.27093300	0.76474900	-0.19017200
C	-8.31369400	-0.91598800	-0.64716200

N	-7.24755400	1.20862700	-0.96689700
N	-7.30955700	-1.36855400	0.12238700
C	-8.28245200	0.38294900	-1.19617700
C	-9.35175400	0.87391300	-2.01930000
C	-9.41619100	-1.80411900	-0.88740300
N	-10.22344000	1.25611600	-2.68543900
N	-10.31414000	-2.51202900	-1.09284500

Cartesian coordinates of DFT-optimized structure of **Ru-dmit** by B3LYP / 6-31G(d,p);LanL2DZ level of theory
Charge = 0 Multiplicity = 1

C	-4.41105600	-0.61623700	-0.29288300
C	-3.34958600	-2.29279800	-1.49820500
C	-4.39155900	0.82290400	0.00649700
C	-5.41363400	-1.49198300	0.11027400
C	-4.35313700	-3.20782300	-1.19425100
H	-2.51265700	-2.55669300	-2.13347100
C	-5.53337000	1.57784900	0.27837600
C	-5.34424100	-2.82786300	-0.28752900
H	-4.32918700	-4.21793500	-1.58751600
C	-5.43266100	2.96703600	0.37685200
C	-3.08540400	2.75137300	-0.09338500
C	-4.18039500	3.55973800	0.17124200
H	-2.10477000	3.16461500	-0.29453000
H	-4.08100700	4.63786200	0.21900600
N	-3.32163700	-1.05503700	-0.98368600
N	-3.17262800	1.40819100	-0.12439800
H	-6.48140200	1.06753100	0.41445600
H	-6.19416300	-1.16051700	0.78536800
C	-6.28464700	-3.85624300	0.27925000
C	-6.59285500	3.86723300	0.70478600
O	-6.82490700	-4.69193000	-0.40597000
O	-6.43617600	4.96597200	1.17780000
O	-7.83742900	3.39128800	0.45613600
O	-6.47340700	-3.76810900	1.60751000
H	-5.77344700	-3.21702300	2.03442800
H	-7.80658300	2.58430500	-0.08282700
N	-1.07841800	0.90029400	-2.15249500
N	-2.16029100	-0.80198400	1.49427500
C	-0.15995900	1.24723300	-2.82249300
C	-2.87535600	-1.39166600	2.22845700
S	-3.88822200	-2.19883000	3.23733600
S	1.11094300	1.71523300	-3.72287300
Ru	-1.61836200	0.06396400	-0.33367800
C	1.05146000	2.99072900	1.56105500
C	2.27837300	2.62874900	1.03574300
C	2.37665800	1.45731500	0.25201800
C	1.18642800	0.72727000	0.03646900
C	-0.08683300	2.22928400	1.25909600

C	3.59772800	0.95157300	-0.33517200
C	1.22338400	-0.56376700	-0.57864000
C	2.45367000	-1.11889000	-0.99772300
C	3.63342000	-0.28370300	-0.93873100
C	2.43590800	-2.46196000	-1.43517100
H	3.35280600	-2.94266200	-1.75639200
C	1.24557000	-3.16344700	-1.41228900
C	0.06665300	-2.51886300	-1.00838400
H	0.95389400	3.86181500	2.19974400
H	3.16293900	3.21884200	1.24614400
H	-1.06276000	2.49128000	1.64958800
H	1.20727400	-4.20668200	-1.70620900
H	-0.87636600	-3.04812400	-0.97667300
N	0.03992400	-1.24139200	-0.63709500
N	-0.03204600	1.14937200	0.48033700
S	5.06014300	1.99020600	-0.28162800
S	5.14440800	-0.89161600	-1.69286400
C	6.29430100	-0.44600400	-0.42145900
C	6.25888200	0.76823500	0.17314800
S	7.38274400	1.03801200	1.50149700
S	7.45997400	-1.60670600	0.20655400
C	8.19380700	-0.53932300	1.42081300
S	9.45470400	-0.97008700	2.37583900

Cartesian coordinates of DFT-optimized structure of **Ru-pdt** by B3LYP / 6-31G(d,p);LanL2DZ level of theory
Charge = 0 Multiplicity = 1

C	-3.85436900	-0.55194000	-0.41671300
C	-2.76979700	-2.31555200	-1.46739100
C	-3.80480600	0.89787700	-0.17909500
C	-4.91421800	-1.37514100	-0.05105600
C	-3.82505900	-3.18228100	-1.19923300
H	-1.89902900	-2.63553300	-2.02692700
C	-4.93522700	1.70153400	-0.02521500
C	-4.86473900	-2.72921900	-0.38507500
H	-3.80901400	-4.20955300	-1.54566300
C	-4.79197100	3.08983400	0.01989000
C	-2.42601100	2.77592300	-0.26632800
C	-3.50744400	3.63097000	-0.11872300
H	-1.41900600	3.14677900	-0.41229100
H	-3.37291700	4.70631200	-0.11119500
N	-2.73417900	-1.05729800	-1.00554800
N	-2.55903900	1.43615500	-0.24452100
H	-5.90831800	1.22916000	0.06277800
H	-5.72858500	-0.98786000	0.55033200
C	-5.87952700	-3.69924500	0.15509200
C	-5.93962000	4.04120600	0.22102900
O	-6.39853000	-4.54827400	-0.53045000
O	-5.77947000	5.15605400	0.65377100
O	-7.17950300	3.59378800	-0.09648200

O	-6.16071300	-3.54367100	1.46070500
H	-5.47365200	-2.99665800	1.91351800
H	-7.13770400	2.76108500	-0.59395800
N	-0.34564300	0.76899300	-2.08470200
N	-1.74959700	-0.73140700	1.53870300
C	0.61965500	1.06678400	-2.71098200
C	-2.53474700	-1.26537400	2.24295400
S	-3.64506400	-1.99543000	3.20826300
S	1.94911500	1.46994700	-3.55596200
Ru	-1.04355300	0.03425900	-0.27970100
C	1.58145900	2.94757100	1.69539400
C	2.83141500	2.51656400	1.29009600
C	2.94723100	1.30959800	0.56365900
C	1.74902700	0.61702600	0.28085800
C	0.44149800	2.21690800	1.33165300
C	4.19501300	0.73788100	0.10021500
C	1.78443000	-0.70068400	-0.27343600
C	3.02268300	-1.31704800	-0.56228600
C	4.23044300	-0.52483500	-0.44535200
C	2.98596900	-2.67902100	-0.93785900
H	3.90581000	-3.20898100	-1.15628200
C	1.77080000	-3.33541300	-0.98526300
C	0.58919000	-2.63113300	-0.71106000
H	1.46842000	3.84871600	2.28826500
H	3.71854300	3.08117100	1.55254400
H	-0.55143800	2.53117100	1.62971300
H	1.71619100	-4.38963500	-1.23414600
H	-0.37357900	-3.12395800	-0.73403900
N	0.58385500	-1.33779100	-0.39915700
N	0.51660900	1.10188600	0.60616200
S	5.66206900	1.74830400	0.24564300
S	5.74735500	-1.24954700	-1.05279600
C	6.89551700	-0.75484500	0.21894100
C	6.85881100	0.53860900	0.77910200
C	8.74569400	-1.22694400	1.44980400
N	7.75533800	0.93474900	1.67657900
N	7.82830300	-1.63664800	0.56322800
C	8.70941600	0.05261300	2.00379100
H	9.52130800	-1.93926500	1.71879100
H	9.45556400	0.38025100	2.72298800

Cartesian coordinates of DFT-optimized structure of **Ru-tdt** by B3LYP / 6-31G(d,p);LanL2DZ level of theory
Charge = 0 Multiplicity = 1

C	-4.06576500	-0.55742400	-0.30975800
C	-3.04176900	-2.31261900	-1.43186800
C	-4.00668300	0.89152100	-0.06961700
C	-5.09936900	-1.38547700	0.11544300
C	-4.07601400	-3.18408800	-1.10401400
H	-2.20607500	-2.62723300	-2.04520400

C	-5.12882600	1.69134200	0.15033600
C	-5.06599800	-2.73803700	-0.22644700
H	-4.07762700	-4.20984400	-1.45526100
C	-4.98748800	3.08006600	0.18970500
C	-2.64072800	2.77391900	-0.23232300
C	-3.71456900	3.62525900	-0.02184200
H	-1.64493600	3.14792800	-0.43575000
H	-3.58314300	4.70100600	-0.02111900
N	-2.98127100	-1.05633500	-0.96718900
N	-2.76805200	1.43353000	-0.20424100
H	-6.09378900	1.21546900	0.29256500
H	-5.87700600	-1.00311900	0.76654800
C	-6.04291700	-3.71365900	0.37016000
C	-6.12452700	4.02741100	0.45703400
O	-6.59926700	-4.56250900	-0.28593700
O	-5.94405000	5.14312900	0.87968100
O	-7.37934000	3.57563400	0.21170300
O	-6.24565900	-3.56389700	1.69085000
H	-5.53451200	-3.01575500	2.10391500
H	-7.36300900	2.74260300	-0.28669900
N	-0.66114700	0.78891900	-2.16511300
N	-1.85346300	-0.74313200	1.51838900
C	0.25586600	1.10653800	-2.85118200
C	-2.59502000	-1.27807500	2.26737700
S	-3.64677800	-2.01009700	3.29563300
S	1.51583700	1.53878200	-3.78343800
Ru	-1.25304600	0.03740700	-0.33248100
C	1.45643500	2.94765100	1.52802700
C	2.68800200	2.51832100	1.06888900
C	2.77406300	1.31341100	0.33574600
C	1.56470200	0.62229100	0.09772200
C	0.30264600	2.21637300	1.21151000
C	4.00254400	0.74243200	-0.17394900
C	1.57662900	-0.68990100	-0.47101500
C	2.80210100	-1.30169900	-0.82014100
C	4.01509300	-0.51591700	-0.73372900
C	2.74921300	-2.65554100	-1.22058100
H	3.66105700	-3.17646900	-1.48875900
C	1.53340300	-3.31192300	-1.22951000
C	0.36484200	-2.61420100	-0.88947200
H	1.36719100	3.84736400	2.12722100
H	3.58729200	3.08070300	1.29181600
H	-0.67645900	2.52793800	1.55450000
H	1.46714700	-4.36083900	-1.49754500
H	-0.59719800	-3.10858700	-0.87898600
N	0.37209300	-1.32711400	-0.55313900
N	0.34727900	1.10455700	0.47877400
S	5.49432700	1.71876600	-0.08366000
S	5.52608900	-1.20755300	-1.38578700
C	6.67685700	-0.78499700	-0.08749400
C	6.66629500	0.49422900	0.48128600
C	8.55774200	-1.36893000	1.31434100

C	8.56188100	-0.09031100	1.88972700
H	9.29205100	-2.10290100	1.63625400
C	9.59577300	0.29087700	2.92256900
H	9.83467800	-0.55297100	3.57877000
H	10.53135800	0.60483400	2.44157800
H	9.25347600	1.12150200	3.54807400
C	7.58920400	0.82757600	1.47714100
C	7.61605200	-1.72416000	0.35073000
H	7.55158600	1.81519300	1.92885700
H	7.61077800	-2.72646600	-0.06711300

Cartesian coordinates of DFT-optimized structure of **Ru-dcdmp@$(TiO_2)_8$** by B3LYP/6-31G(d,p);LanL2DZ level of theory
Charge = 0 Multiplicity = 1

C	-0.18267500	-0.17435500	0.80151600
C	-1.14875900	-2.12543200	1.58419800
C	-0.33748700	1.26819600	0.59596800
C	0.91885300	-0.86817300	0.31818400
C	-0.08295400	-2.89509400	1.12290300
H	-1.97496300	-2.57323200	2.12143700
C	0.73272700	2.11646100	0.33004100
C	0.91460000	-2.25922900	0.37729000
H	-0.06798000	-3.97030100	1.26277400
C	0.48272200	3.46568600	0.07827000
C	-1.82861200	3.05516900	0.59382000
C	-0.81817100	3.95452700	0.28480400
H	-2.84528500	3.38005500	0.77587500
H	-1.02987000	5.01259600	0.18089700
N	-1.26498700	-0.81286600	1.31752500
N	-1.61698100	1.72302500	0.64263600
H	1.74012100	1.72419200	0.39830000
H	1.70801000	-0.34244500	-0.19748900
C	1.86462000	-3.04954000	-0.45228000
C	1.50029700	4.40591700	-0.42658300
O	2.25648800	-4.15579300	-0.24689700
O	1.46384600	5.60218500	-0.40551800
O	2.59490500	3.77518600	-1.09842000
O	2.25855400	-2.34327000	-1.62776700
H	1.47067800	-1.83822000	-2.03458200
H	3.18198600	4.51273600	-1.37628500
N	-3.80614500	0.83670900	2.43997900
C	-4.82889300	1.06325500	3.00508400
S	-6.23679400	1.36218300	3.75275500
N	-2.23670900	-0.38296900	-1.22249000
C	-1.37352200	-0.61215700	-1.99326600
S	-0.15407000	-0.89935000	-3.06524900
Ru	-3.03172800	0.22455800	0.63029200
C	-5.78775200	3.01936500	-1.34706500
C	-7.01573300	2.52713900	-0.94487200
C	-7.07359900	1.30982600	-0.22902000

C	-5.84479700	0.67037700	0.04691000
C	-4.61501700	2.34418700	-0.98095400
C	-8.29064800	0.67695700	0.23581300
C	-5.81837900	-0.65969400	0.57200100
C	-7.02629800	-1.33602800	0.85397900
C	-8.26659600	-0.59231000	0.76258700
C	-6.92908300	-2.70202900	1.20306600
H	-7.82171300	-3.27850400	1.41669500
C	-5.68629200	-3.30464200	1.23455800
C	-4.53884500	-2.54255900	0.97041500
H	-5.71774900	3.92701200	-1.93637000
H	-7.92689800	3.05367400	-1.20435400
H	-3.63869000	2.70946600	-1.27442900
H	-5.58361900	-4.35931300	1.46487400
H	-3.55682800	-2.99443200	0.98398500
N	-4.59031400	-1.24519300	0.68055800
N	-4.63703400	1.22364500	-0.26016900
S	-9.79538500	1.63611500	0.11841100
S	-9.73946900	-1.39666700	1.38147900
C	-10.93212200	-0.91628200	0.15813800
C	-10.95619800	0.39273400	-0.38752000
C	-12.83085000	-1.42836400	-0.99711300
N	-11.90198200	0.77050900	-1.23356700
N	-11.85511000	-1.81183500	-0.15703600
C	-12.85438400	-0.12606000	-1.54028600
C	-13.89193600	0.29138400	-2.44055300
C	-13.84381800	-2.39288300	-1.32093200
N	-14.73604500	0.61393300	-3.17062600
N	-14.66899900	-3.16316200	-1.59534100
Ti	5.50733200	-1.88948800	1.59446300
Ti	5.47629200	1.41098400	1.78962900
Ti	4.12470700	-1.33631200	-1.51295300
Ti	3.69845400	1.92253200	-1.20671700
O	5.22283200	-2.60977300	-2.18392700
O	5.25258900	2.46825600	-1.87290800
O	5.55665200	-0.28203500	2.46709400
O	5.43759400	2.57189000	2.92643200
O	4.16824300	-1.54909100	0.18592100
O	4.00588200	1.63399700	0.46942900
O	5.06691000	-3.12665400	2.55008000
O	3.29713100	0.33741600	-1.92743400
Ti	9.13317200	-2.01771200	0.14277100
Ti	9.04650800	1.41347600	0.23908100
Ti	6.83596800	-2.04381500	-1.42768700
Ti	6.70254200	1.41432300	-1.28355500
O	8.52513800	-2.18898600	-1.75550200
O	8.37087100	1.60888400	-1.63752500
O	9.60393200	-0.29719100	0.57929600
O	9.97464800	2.61175900	0.81538400
O	7.08976000	-2.21269700	0.37756900
O	7.01091500	1.55423100	0.51190100
O	10.05916400	-3.20223600	0.74949300

O	6.06627000	-0.32466500	-1.52521400

Cartesian coordinates of DFT-optimized structure of **Ru-dmit@$(TiO_2)_8$** by B3LYP / 6-31G(d,p);LanL2DZ level of theory

Charge = 0 Multiplicity = 1

Ti	-7.98278600	-0.68338700	-0.77717200
Ti	-7.21656000	2.22803100	0.80907600
Ti	-4.84135500	-0.19385600	-1.85613900
Ti	-4.11621200	2.75214500	-0.84788200
O	-4.37326700	-1.97566500	-1.85620900
O	-3.38421100	2.11886900	0.66347700
O	-8.24268200	0.84923500	0.17742800
O	-8.07238500	3.40364400	1.52256100
O	-6.54097900	-0.14254300	-2.02353400
O	-5.79056900	2.88203200	-0.43943500
O	-9.31831000	-1.22051200	-1.51980400
O	-3.94173800	1.40033200	-2.06328300
Ti	-6.65281800	-2.96399800	2.10643800
Ti	-5.85381400	-0.00982500	3.72042300
Ti	-5.14537400	-2.30490700	-0.14049100
Ti	-4.36104200	0.78416800	1.51374700
O	-4.83445200	-3.25407500	1.24691000
O	-3.98363300	-0.00287800	2.98935700
O	-6.64131300	-1.62894800	3.35370500
O	-6.04735200	0.58438100	5.21292600
O	-6.91454900	-2.14644400	0.21164300
O	-6.00851300	1.25894000	2.10247200
O	-7.53059100	-4.27639300	2.45900300
O	-4.71574300	-0.43527400	0.08874200
C	-0.16456500	0.77342000	-1.16485800
C	0.49672600	2.01805800	0.67713000
C	-0.00129100	-0.49593200	-1.88727500
C	-0.97338500	1.80811400	-1.62277500
C	-0.25817900	3.11259900	0.27708100
H	1.06404000	2.04202400	1.59749300
C	-1.00973000	-1.11123400	-2.63199500
C	-0.96372400	3.01234400	-0.92367300
H	-0.26995400	4.01877700	0.87202100
C	-0.76856200	-2.36258300	-3.20368700
C	1.41755800	-2.31113100	-2.19118900
C	0.46928200	-2.97056700	-2.96002400
H	2.36820600	-2.76567700	-1.94230500
H	0.68049800	-3.94592000	-3.38300700
N	0.62992000	0.90919900	-0.06776800
N	1.21139300	-1.07104300	-1.70689900
H	-1.95796600	-0.60432200	-2.76847000
H	-1.53764800	1.69888200	-2.53956800
C	-1.68148600	4.19432600	-1.48212900
C	-1.71842800	-3.05214700	-4.15701400

O	-2.94043500	4.26846900	-1.46217000
O	-1.27456900	-3.73651800	-5.05004100
O	-3.03378700	-2.83564900	-4.01975900
O	-0.98363700	5.14666500	-1.99110700
H	0.00882800	4.86854900	-2.09548900
H	-3.33159100	-2.54229600	-3.12303200
N	2.60005300	-1.65631600	0.71187900
C	3.36072700	-2.36823100	1.28858700
S	4.41314100	-3.32482500	2.06672800
N	2.56040500	1.72646100	-1.73649100
C	2.28688100	2.81150500	-2.10405600
S	1.93149900	4.33986500	-2.63301600
Ru	2.55876600	-0.00630800	-0.52962300
C	5.73029600	-1.78501500	-2.94391000
C	6.79119800	-1.81240800	-2.05753200
C	6.64766100	-1.21630300	-0.78451100
C	5.39998000	-0.62134200	-0.49060900
C	4.51013900	-1.21854000	-2.54989600
C	7.67495700	-1.17821000	0.23187500
C	5.21732500	0.15057400	0.69916500
C	6.27499300	0.28074300	1.62867700
C	7.49088900	-0.46769900	1.39554100
C	6.06186200	1.13933000	2.72965600
H	6.84280000	1.28556000	3.46687600
C	4.85692600	1.80389100	2.84260800
C	3.85894300	1.59744700	1.87907800
H	5.82396300	-2.20509100	-3.93929900
H	7.73372800	-2.26528700	-2.34297800
H	3.65497000	-1.19448600	-3.21441800
H	4.66765400	2.48210300	3.66731100
H	2.91106100	2.11059900	1.95380600
N	4.01483500	0.78419900	0.83846100
N	4.33684000	-0.68399000	-1.34065100
S	9.17934400	-2.11588300	-0.04349200
S	8.75014900	-0.46754000	2.67437000
C	10.18133500	-0.22547600	1.65853300
C	10.36240900	-0.92039900	0.51265000
S	11.76439500	-0.49749700	-0.46431400
S	11.37005800	1.01757200	2.03374100
C	12.42396100	0.73600800	0.63131500
S	13.82605600	1.53969500	0.36505500

Cartesian coordinates of DFT-optimized structure of **Ru-pdt@(TiO$_2$)$_8$** by B3LYP / 6-31G(d,p);LanL2DZ level of theory
Charge = 0 Multiplicity = 1

Ti	-5.04616000	-1.88235100	-1.62507200
Ti	-4.95836200	1.41388900	-1.88848700
Ti	-3.66244700	-1.29346100	1.47643100
Ti	-3.18666600	1.95573400	1.10450300
O	-4.79158800	-2.52866900	2.16969200

O	-4.73522300	2.54115800	1.75175700
O	-5.06579200	-0.29192700	-2.52925500
O	-4.90224100	2.54856000	-3.05083700
O	-3.70572500	-1.54013900	-0.21742500
O	-3.48894300	1.64299000	-0.56805500
O	-4.63077600	-3.14605000	-2.55692700
O	-2.81662300	0.37807700	1.85876200
Ti	-8.67887600	-1.92008000	-0.18766400
Ti	-8.53533200	1.50610800	-0.35577800
Ti	-6.38912000	-1.94982500	1.39285300
Ti	-6.19923400	1.49990700	1.17772200
O	-8.08251200	-2.06135800	1.71573400
O	-7.86647900	1.73025400	1.51888200
O	-9.12079600	-0.20175400	-0.66190800
O	-9.44195400	2.70728700	-0.96052000
O	-6.63903300	-2.15354800	-0.40996300
O	-6.49691000	1.60741900	-0.62180100
O	-9.62365600	-3.10193000	-0.77112000
O	-5.59395200	-0.24290000	1.45823900
C	0.70877200	-0.23221400	-0.76411700
C	1.66473700	-2.20776600	-1.49597600
C	0.87952500	1.21059500	-0.57557400
C	-0.41457400	-0.90305800	-0.29928300
C	0.57578000	-2.95520200	-1.05297300
H	2.49781000	-2.67507400	-2.00516700
C	-0.18381900	2.07978500	-0.35533000
C	-0.43090500	-2.29464000	-0.34149500
H	0.54841800	-4.03184000	-1.17913300
C	0.08039900	3.43021400	-0.12208400
C	2.39873400	2.97456000	-0.56553400
C	1.39445100	3.89469700	-0.30261000
H	3.42520100	3.27958300	-0.72560000
H	1.61975700	4.95131000	-0.21358000
N	1.79458500	-0.89346400	-1.24361100
N	2.16760800	1.64447100	-0.59291400
H	-1.19491000	1.70128700	-0.44584300
H	-1.20657200	-0.35849000	0.19211500
C	-1.41417600	-3.05813000	0.47309500
C	-0.93428200	4.39373200	0.33821100
O	-1.82161200	-4.15975000	0.27261800
O	-0.87929300	5.58927700	0.30274800
O	-2.05953700	3.78944300	0.98520900
O	-1.82284900	-2.32923400	1.63041500
H	-1.03143200	-1.83995500	2.05469400
H	-2.64378600	4.53911800	1.23460100
N	4.39260000	0.70949400	-2.30588600
C	5.42920200	0.92403600	-2.85050300
S	6.84982100	1.20897800	-3.57709900
N	2.69913700	-0.45065400	1.31629800
C	1.82032100	-0.67248500	2.07068000
S	0.57943800	-0.95192900	3.12159100
Ru	3.55722800	0.12863300	-0.51830100

C	6.27345300	2.90559300	1.53653000
C	7.50976100	2.38232300	1.20586900
C	7.58077200	1.15443900	0.50938100
C	6.35415700	0.53698000	0.17705500
C	5.10737300	2.24791100	1.12098000
C	8.81033900	0.49365200	0.12294400
C	6.32904500	-0.79748200	-0.33592300
C	7.53814200	-1.49735100	-0.54874000
C	8.78888400	-0.78206300	-0.39362700
C	7.43286800	-2.86355800	-0.89496600
H	8.32676400	-3.45502900	-1.05563900
C	6.18280100	-3.44364000	-0.99064000
C	5.03733800	-2.66006000	-0.78855300
H	6.19171400	3.82284500	2.10935900
H	8.41890000	2.89057600	1.50568500
H	4.12521000	2.63531300	1.36172800
H	6.07266800	-4.49746800	-1.22170900
H	4.04962400	-3.09503400	-0.84933200
N	5.09757400	-1.36290900	-0.50036900
N	5.14371800	1.11657600	0.41813800
S	10.32921300	1.41337000	0.31473400
S	10.27993400	-1.61513300	-0.91647600
C	11.40058000	-1.16241600	0.39650500
C	11.42178100	0.14241300	0.92746600
C	13.15697800	-1.72301800	1.72309100
N	12.29832400	0.50185100	1.85890700
N	12.25654100	-2.09377100	0.80282300
C	13.17774200	-0.43074400	2.24899000
H	13.87157200	-2.47677400	2.04285100
H	13.90916200	-0.13443200	2.99615900

Cartesian coordinates of DFT-optimized structure of **Ru-tdt@$(TiO_2)_8$** by B3LYP / 6-31G(d,p);LanL2DZ level of theory
Charge = 0 Multiplicity = 1

Ti	5.24405000	1.86537100	-1.60398400
Ti	5.16967700	-1.43504000	-1.81926500
Ti	3.77737400	1.32287800	1.46954000
Ti	3.32457700	-1.93491000	1.13643300
O	4.88929300	2.56672900	2.17471200
O	4.85749000	-2.50675700	1.83190800
O	5.29027900	0.26130400	-2.48263500
O	5.14693500	-2.58708300	-2.96560500
O	3.86867900	1.54244700	-0.22606700
O	3.66817500	-1.64847700	-0.53271200
O	4.85199700	3.11397100	-2.56593600
O	2.93009600	-0.34665400	1.85612400
Ti	8.83704100	1.93413300	-0.07347600
Ti	8.70684800	-1.49431900	-0.19668000
Ti	6.50684400	1.97986200	1.44645000
Ti	6.33336700	-1.47182800	1.27780100

O	8.19128600	2.10062600	1.81127400
O	7.99247500	-1.69243100	1.66362000
O	9.29612800	0.21068600	-0.51174600
O	9.63261800	-2.70138700	-0.75957600
O	6.80371000	2.15895000	-0.35219000
O	6.67633500	-1.60641500	-0.51218200
O	9.79389300	3.11065800	-0.64834000
O	5.71644100	0.27235800	1.51619600
C	-0.53347500	0.20445800	-0.85257500
C	-1.47258200	2.16029700	-1.65402800
C	-0.70606700	-1.23391300	-0.63497100
C	0.57628300	0.88795200	-0.37396100
C	-0.39676200	2.92004300	-1.20019500
H	-2.29216300	2.61458400	-2.19567900
C	0.35375300	-2.09656100	-0.37656800
C	0.59237600	2.27818600	-0.44808200
H	-0.36808000	3.99357500	-1.35016800
C	0.08692200	-3.44277400	-0.12151400
C	-2.22324000	-2.99979500	-0.61846400
C	-1.22307200	-3.91267800	-0.31795600
H	-3.24605400	-3.30937600	-0.79253400
H	-1.44873000	-4.96769600	-0.21245000
N	-1.60768600	0.85169500	-1.37570200
N	-1.99325000	-1.66986800	-0.66780700
H	1.36578200	-1.71793700	-0.45642700
H	1.35515000	0.35601000	0.15109800
C	1.55340100	3.06187800	0.37324300
C	1.09391400	-4.39466300	0.37620900
O	1.96224900	4.16062900	0.16028100
O	1.04110800	-5.59095200	0.36682000
O	2.20889600	-3.77460300	1.02677700
O	1.93640000	2.35797300	1.55535700
H	1.13262500	1.88315200	1.97514700
H	2.79096800	-4.51736300	1.30052800
N	-4.17993200	-0.79688200	-2.44825000
C	-5.19925800	-1.04358400	-3.01167200
S	-6.59379200	-1.37319500	-3.76840600
N	-2.57132100	0.47831400	1.17219000
C	-1.71564400	0.72632800	1.94447400
S	-0.50761900	1.04416500	3.02320800
Ru	-3.38692100	-0.15775500	-0.66369600
C	-6.12052400	-2.88662200	1.42923300
C	-7.35458400	-2.37529100	1.07313900
C	-7.42246500	-1.16618000	0.34503100
C	-6.19401600	-0.55552200	0.00453100
C	-4.95211900	-2.23627400	1.00813600
C	-8.65013700	-0.51851500	-0.06400600
C	-6.16769000	0.76087100	-0.55326300
C	-7.37735000	1.44769000	-0.80485900
C	-8.62737800	0.73971800	-0.62513600
C	-7.27344300	2.79680500	-1.21142400
H	-8.17028800	3.37299500	-1.40744800

C	-6.02490600	3.37700800	-1.32198100
C	-4.87873300	2.60801400	-1.07205800
H	-6.04118900	-3.78871500	2.02609000
H	-8.26750500	-2.87599000	1.37422000
H	-3.97149300	-2.61362100	1.26996600
H	-5.91532000	4.41932400	-1.60084400
H	-3.89258000	3.04478100	-1.14193900
N	-4.93649800	1.32494200	-0.72655700
N	-4.98461700	-1.12489100	0.27401100
S	-10.18550000	-1.40299800	0.14035400
S	-10.13527900	1.52271500	-1.16774500
C	-11.21415800	1.16730800	0.21066400
C	-11.23928900	-0.10998800	0.78221200
C	-12.95110700	1.86206300	1.74068200
C	-12.99042900	0.58580200	2.32020200
H	-13.61593800	2.63822000	2.11067900
C	-13.96932800	0.26718900	3.42503300
H	-14.10220000	1.12035400	4.09884300
H	-14.95627300	0.02107000	3.01229200
H	-13.63974200	-0.58940000	4.02150500
C	-12.10606800	-0.38859800	1.84300200
C	-12.06150900	2.16117000	0.71083800
H	-12.09575900	-1.37694600	2.29443300
H	-12.02734600	3.16134900	0.28939400

Cartesian coordinates of DFT-optimized structure of **N3 dye** by B3LYP / 6-31G(d,p);LanL2DZ level of theory
Charge = 0 Multiplicity = 1

C	-2.74500800	0.82285600	-0.32240700
C	-1.39319900	2.69834000	-0.58308600
C	-2.77071100	-0.64944500	-0.36231700
C	-3.89555900	1.61361300	-0.35865100
C	-2.49359000	3.54312300	-0.62553000
C	-3.84430800	-1.42569400	0.06363100
C	-3.77590700	2.99793300	-0.49971500
H	-2.37077500	4.61162000	-0.75868400
C	-3.78755600	-2.81080100	-0.10453000
C	-1.65918400	-2.51785200	-1.16155700
C	-2.72466400	-3.35133100	-0.82977000
H	-0.77663900	-2.90333700	-1.65839200
H	-2.69662700	-4.41083300	-1.05883900
N	-1.50714700	1.37202600	-0.38792400
N	-1.63360000	-1.21476700	-0.84975500
H	-4.68114100	-0.96907000	0.58025800
H	-4.86580800	1.13487500	-0.27330500
C	-4.95047100	3.94066300	-0.50958200
C	-4.82239100	-3.71099100	0.51746200
O	-4.84225300	5.10012700	-0.19668400
O	-5.35290000	-4.61146000	-0.08787100

O	-5.10665300	-3.42896600	1.80080000
O	-6.14948500	3.42840300	-0.87689800
H	-6.04953600	2.54445400	-1.26656600
H	-4.42571200	-2.83842200	2.20477100
H	-0.38577800	3.07361500	-0.71356500
N	-0.72639000	-0.56849700	1.68919100
N	0.72711100	0.51225900	-2.03937200
C	-1.50138500	-1.03950400	2.44794100
C	1.04856500	0.88341700	-3.11816100
S	1.50141900	1.38873800	-4.59792900
S	-2.59685700	-1.68763300	3.48561000
Ru	0.05290000	-0.00766500	-0.18006300
N	1.68954400	-1.33926300	-0.06563000
N	1.53632600	1.16346400	0.65455700
C	1.68114200	-2.66942900	-0.24581700
H	0.70700600	-3.13710000	-0.27414900
C	2.83877500	-3.42608900	-0.38242500
H	2.77707300	-4.49662900	-0.53448400
C	4.09226000	-1.40073900	-0.07995900
H	5.05042200	-0.90160700	-0.00293100
C	2.89060700	-0.70896500	0.06774000
C	2.80473200	0.69459000	0.49800000
C	3.91147000	1.46821900	0.85400400
H	4.91733100	1.09891800	0.70257100
C	2.40641000	3.15686800	1.66496500
H	2.22844900	4.11062700	2.14798200
C	1.35248100	2.34838900	1.26737900
H	0.32397600	2.63881300	1.43853200
C	5.38040500	-3.47736100	-0.48243300
O	6.45941000	-2.93086200	-0.39958200
O	5.22984200	-4.79649200	-0.72687600
H	6.12812400	-5.16760300	-0.81631600
C	4.07291900	-2.77264800	-0.32451800
C	4.84551500	3.61294600	1.82817500
O	4.70146000	4.70020600	2.34389400
O	6.05283700	3.07261000	1.55273000
H	6.72133600	3.72223300	1.84175800
C	3.71510900	2.72387800	1.42744200

Cartesian coordinates of DFT-optimized structure of **N3 dye** by CAM-B3LYP / 6-31G(d,p);LanL2DZ level of theory
Charge = 0 Multiplicity = 1

C	-2.74866800	0.82983800	-0.37122700
C	-1.38428900	2.68346400	-0.60252300
C	-2.78507600	-0.64398800	-0.40235600
C	-3.88941600	1.62183100	-0.43525400
C	-2.47317400	3.53405500	-0.67179500
C	-3.86008400	-1.39979500	0.03324000
C	-3.75346700	2.99796400	-0.57640300
H	-2.34270000	4.60123700	-0.80221300

C	-3.80804600	-2.78168400	-0.11029400
C	-1.68908000	-2.51938900	-1.16659100
C	-2.75263900	-3.33999200	-0.81817100
H	-0.80904300	-2.91768900	-1.65608400
H	-2.72985100	-4.40375400	-1.02292200
N	-1.51522500	1.36549800	-0.41205300
N	-1.66025700	-1.21670100	-0.88347200
H	-4.69288400	-0.93099900	0.54402500
H	-4.86375300	1.14988200	-0.37093900
C	-4.91908600	3.94691000	-0.62141800
C	-4.84668500	-3.66266500	0.52486700
O	-4.80811300	5.10147200	-0.31344500
O	-5.37184900	-4.57009800	-0.06454800
O	-5.13361800	-3.35133500	1.79058200
O	-6.10218100	3.44153000	-1.01596700
H	-6.00110300	2.55738000	-1.39901100
H	-4.44857400	-2.76650700	2.19436100
H	-0.37101600	3.04892400	-0.71088500
N	-0.78538200	-0.59976700	1.65902700
N	0.75920200	0.53026300	-2.02038500
C	-1.56633700	-1.04293700	2.41414900
C	1.24204900	0.91305500	-3.02270000
S	1.91239100	1.44321500	-4.40745800
S	-2.69053400	-1.65999800	3.44077000
Ru	0.03089100	-0.02418300	-0.18850600
N	1.67160700	-1.35535400	-0.07351600
N	1.50794300	1.13812300	0.67798900
C	1.66788500	-2.67293600	-0.28755800
H	0.69621300	-3.14198600	-0.33916700
C	2.82609600	-3.42000000	-0.42922300
H	2.77399900	-4.48598200	-0.60945600
C	4.06160600	-1.40448400	-0.06528900
H	5.01707300	-0.90552500	0.03264500
C	2.85899700	-0.72753800	0.08240800
C	2.76648000	0.67190900	0.53459800
C	3.86712400	1.43202200	0.91337800
H	4.87465000	1.06409600	0.77542400
C	2.36032800	3.11314000	1.71116400
H	2.18076400	4.06400300	2.19768600
C	1.31452400	2.31228100	1.29052700
H	0.28383000	2.60278900	1.44514600
C	5.35752000	-3.46068000	-0.49645400
O	6.42632000	-2.91347400	-0.38053400
O	5.21760100	-4.76389900	-0.77800300
H	6.11517600	-5.13249400	-0.86490100
C	4.04733400	-2.76419200	-0.33981500
C	4.78834200	3.55564600	1.92056700
O	4.63737700	4.63339400	2.43985900
O	5.98962600	3.01612700	1.66510000
H	6.65813400	3.65651800	1.96829000
C	3.66170300	2.67624800	1.49246800

Cartesian coordinates of DFT-optimized structure of **N3 dye** by M062X/ 6-31G(d,p);LanL2DZ level of theory
Charge = 0 Multiplicity = 1

C	-2.79210100	0.85661700	-0.56787500
C	-1.36052300	2.66800200	-0.76333100
C	-2.87395500	-0.61989300	-0.53670700
C	-3.90741000	1.67686200	-0.71990400
C	-2.42043400	3.54917200	-0.91809600
C	-3.99236000	-1.31375000	-0.09829500
C	-3.71865500	3.04533900	-0.88826700
H	-2.25582500	4.61042200	-1.06335300
C	-3.96945200	-2.70518500	-0.14625500
C	-1.78953800	-2.57380200	-1.10302300
C	-2.89088000	-3.34449500	-0.74388000
H	-0.89069600	-3.02498500	-1.50906900
H	-2.88816200	-4.42264000	-0.85703500
N	-1.54540200	1.35994200	-0.55646500
N	-1.75088500	-1.25195300	-0.93706100
H	-4.83310200	-0.78928600	0.34397400
H	-4.89816700	1.23324300	-0.69769300
C	-4.85427300	4.02611400	-1.03223100
C	-5.07717700	-3.51912400	0.46990600
O	-4.72474300	5.18528200	-0.75787000
O	-5.52911400	-4.49253200	-0.06980500
O	-5.50838000	-3.06484200	1.64697900
O	-6.02504100	3.53644100	-1.47634000
H	-5.92309700	2.64020500	-1.82955600
H	-4.85504400	-2.45138100	2.05930900
H	-0.32777400	2.99697900	-0.81394100
N	-1.02376200	-0.53287300	1.65022000
N	0.94324000	0.41864400	-1.97691600
C	-1.91706300	-0.84574800	2.34383500
C	1.98842600	0.63619400	-2.47884400
S	3.45906500	0.92484700	-3.11862200
S	-3.19428900	-1.26982000	3.29180500
Ru	-0.03235000	-0.06559700	-0.17567800
N	1.61405700	-1.42691800	0.07503500
N	1.45473000	1.10875600	0.71615500
C	1.60529900	-2.75003300	-0.09105000
H	0.62947900	-3.22012900	-0.08751500
C	2.76647000	-3.49608400	-0.24939900
H	2.72138500	-4.56935600	-0.38697600
C	4.00362500	-1.45003700	-0.01947900
H	4.95692100	-0.93570200	0.00051400
C	2.79767200	-0.78211300	0.15884000
C	2.70832600	0.64069100	0.55484000
C	3.81829300	1.42622800	0.85470700
H	4.82489100	1.06491300	0.68729900
C	2.31571600	3.13795100	1.62523800
H	2.14728600	4.11143100	2.07072900

C	1.26198300	2.30870500	1.27312600
H	0.22803600	2.59325700	1.43634600
C	5.29261000	-3.51941000	-0.42679100
O	6.36086200	-2.96968100	-0.35118200
O	5.14175900	-4.82677900	-0.68304000
H	6.03443800	-5.19951200	-0.79465600
C	3.98283600	-2.82181100	-0.23842800
C	4.74649500	3.60884700	1.72534300
O	4.59783500	4.71327700	2.18124500
O	5.94130500	3.05812000	1.47268100
H	6.61751900	3.71695100	1.71116000
C	3.61260200	2.69542900	1.38122500

Cartesian coordinates of DFT-optimized structure of **N3 dye** by WB97XD/ 6-31G(d,p);LanL2DZ level of theory
Charge = 0 Multiplicity = 1

C	-2.76628500	0.90093000	-0.47673700
C	-1.34031400	2.71823700	-0.64969300
C	-2.83652300	-0.57272000	-0.50600200
C	-3.88233200	1.72423300	-0.58465900
C	-2.40232300	3.59972000	-0.75779800
C	-3.94955100	-1.30561900	-0.12646200
C	-3.70152500	3.09802700	-0.71495400
H	-2.23365800	4.66335600	-0.87740900
C	-3.91127900	-2.69103800	-0.25406000
C	-1.72614800	-2.47756200	-1.18467100
C	-2.82222500	-3.27689300	-0.88896200
H	-0.82085800	-2.89096400	-1.61369600
H	-2.80347200	-4.34386400	-1.07876500
N	-1.51678000	1.40327600	-0.46856800
N	-1.69628800	-1.17078600	-0.91607600
H	-4.80162800	-0.81774900	0.33409200
H	-4.87247000	1.28029900	-0.55728800
C	-4.84207600	4.07885400	-0.79911500
C	-5.00752500	-3.54983100	0.31871400
O	-4.72642100	5.21775700	-0.43688900
O	-5.51881500	-4.44318900	-0.30482500
O	-5.36096100	-3.23560800	1.56522400
O	-6.00490000	3.61934100	-1.29367600
H	-5.89632900	2.75060700	-1.70286300
H	-4.69429700	-2.65514400	1.99894700
H	-0.31188700	3.05266200	-0.71695700
N	-0.94953700	-0.54005500	1.65978700
N	0.86586100	0.46802100	-1.95183300
C	-1.79632000	-0.95726100	2.36052200
C	1.81232100	0.71919300	-2.61227600
S	3.12729700	1.06160300	-3.50565300
S	-3.00432400	-1.53151300	3.31426300
Ru	-0.02591800	-0.02170600	-0.15335500
N	1.56062400	-1.39469900	0.06175100

N	1.46619300	1.11663200	0.71616800
C	1.51356700	-2.72488200	-0.04943800
H	0.52620800	-3.16652100	-0.02143200
C	2.64937600	-3.50875400	-0.18208900
H	2.56459600	-4.58396500	-0.27862600
C	3.94839200	-1.50077500	-0.01410700
H	4.91709400	-1.01675400	0.00627000
C	2.76701300	-0.78751000	0.13930900
C	2.71390400	0.63549900	0.52603600
C	3.83685400	1.40688900	0.80408800
H	4.83361100	1.03340300	0.60890200
C	2.37682000	3.12266100	1.63010000
H	2.22495600	4.09114900	2.09175100
C	1.30589300	2.31097700	1.29924700
H	0.28280600	2.60663500	1.49814200
C	5.17686900	-3.61761100	-0.36208800
O	6.26373200	-3.09610100	-0.30652300
O	4.99081900	-4.92738000	-0.57593900
H	5.87136100	-5.32495900	-0.67369700
C	3.88907400	-2.87659800	-0.19614300
C	4.81585000	3.57405900	1.66098000
O	4.69400800	4.67754500	2.13418700
O	5.99764100	3.01828500	1.36240100
H	6.68244100	3.66941300	1.58566500
C	3.66399100	2.67512400	1.34468100

Cartesian coordinates of DFT-optimized structure of **N3 dye** by PBEPBE/ 6-31G(d,p);LanL2DZ level of theory
Charge = 0 Multiplicity = 1

C	-2.72807700	0.85519000	-0.30430600
C	-1.39600100	2.74394800	-0.70397800
C	-2.72664300	-0.61450300	-0.31036500
C	-3.88891600	1.64019500	-0.31377000
C	-2.51135400	3.57584000	-0.72782900
C	-3.78690000	-1.42292700	0.11164900
C	-3.79117700	3.02736300	-0.51240400
H	-2.41057900	4.64783400	-0.91330300
C	-3.69339900	-2.81335900	-0.05691600
C	-1.55218600	-2.47242000	-1.10397500
C	-2.61111400	-3.32952700	-0.78766400
H	-0.64337300	-2.83432500	-1.59149900
H	-2.56306200	-4.39517600	-1.02525600
N	-1.48520100	1.41693600	-0.43853000
N	-1.55894000	-1.16419600	-0.77622500
H	-4.64605500	-0.98373700	0.62595500
H	-4.85520300	1.14883000	-0.15982200
C	-4.96399600	3.97044800	-0.49641600
C	-4.70487500	-3.73917500	0.56820900
O	-4.83437600	5.16258800	-0.28463500
O	-5.21914500	-4.66496400	-0.03412700

O	-4.99365900	-3.45447000	1.86090700
O	-6.19888400	3.43442800	-0.72135100
H	-6.11141200	2.50772600	-1.03159500
H	-4.32148500	-2.81652200	2.23578600
H	-0.38961300	3.12050300	-0.89956000
N	-0.71799800	-0.36894500	1.69954500
N	0.71747100	0.40071000	-2.04426900
C	-1.49183200	-0.90099500	2.44460000
C	0.96782700	0.24569900	-3.20971000
S	1.33123800	0.09118000	-4.78183000
S	-2.55130400	-1.60492000	3.47366600
Ru	0.05531000	0.08066400	-0.16618500
N	1.65058600	-1.23969500	0.07269200
N	1.51981700	1.29942500	0.55569400
C	1.61492200	-2.59086800	0.06974800
H	0.62136800	-3.03851900	0.11805300
C	2.76283200	-3.38086600	0.01540400
H	2.68167400	-4.46942300	0.00424200
C	4.06463300	-1.34755800	0.04581700
H	5.04194100	-0.85886100	0.05262700
C	2.87422400	-0.61488500	0.12372600
C	2.80235400	0.82157100	0.40895100
C	3.91456700	1.63451200	0.66360600
H	4.92638400	1.25309000	0.51338100
C	2.41028800	3.38289800	1.37922200
H	2.24123900	4.37947700	1.79408800
C	1.34642900	2.53802100	1.08495300
H	0.31201600	2.83275300	1.27076800
C	5.31180700	-3.48502900	-0.12165600
O	6.41149100	-2.94801600	-0.12968500
O	5.13136200	-4.83203300	-0.19411500
H	6.03841900	-5.21310400	-0.25169600
C	4.01738600	-2.74537000	-0.03051400
C	4.85375500	3.87331200	1.41831900
O	4.71471600	5.01091300	1.84868500
O	6.06790100	3.31694000	1.14510100
H	6.72733200	4.01517900	1.36520800
C	3.72584800	2.94218200	1.13309400
